# PhiReX 2.0: A Programmable and Red Light-Regulated CRISPR-dCas9 System for the Activation of Endogenous Genes in *Saccharomyces cerevisiae*

**DOI:** 10.1021/acssynbio.2c00517

**Published:** 2023-04-04

**Authors:** Fabian Machens, Guangyao Ran, Ciaran Ruehmkorff, Julie Meyer auf der Heyde, Bernd Mueller-Roeber, Lena Hochrein

**Affiliations:** † Department of Molecular Biology, University of Potsdam, Potsdam 14476, Germany; ‡ Max Planck Institute of Molecular Plant Physiology, Potsdam 14476, Germany; § Center of Plant Systems Biology and Biotechnology (CPSBB), Plovdiv 4000, Bulgaria

**Keywords:** optogenetics, CRISPR/Cas9, light induction, metabolic engineering, gene expression, transcription factor, transcriptional regulation

## Abstract

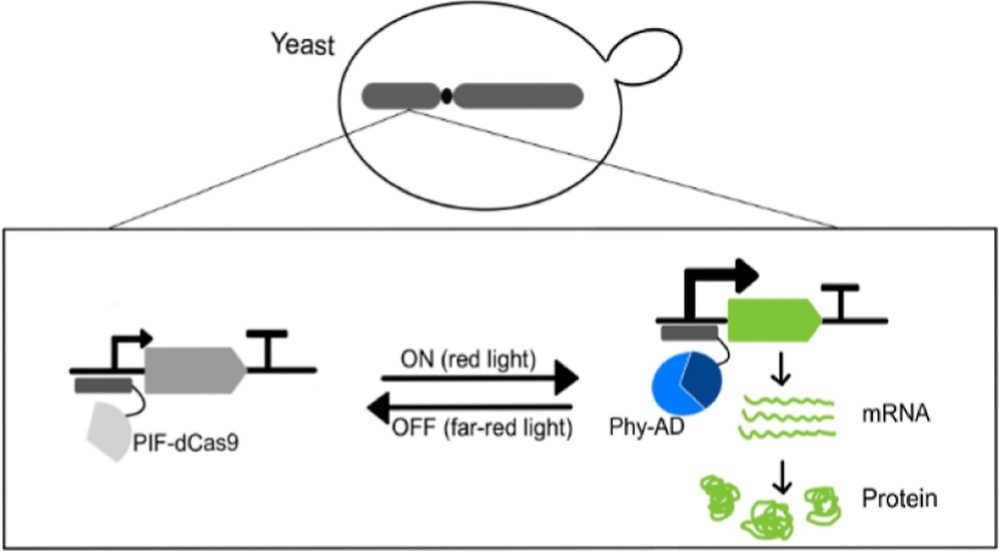

Metabolic engineering approaches do not exclusively require fine-tuning of heterologous genes but oftentimes also modulation or even induction of host gene expression, *e.g.*, in order to rewire metabolic fluxes. Here, we introduce the programmable red light switch PhiReX 2.0, which can rewire metabolic fluxes by targeting endogenous promoter sequences through single-guide RNAs (sgRNAs) and activate gene expression in *Saccharomyces cerevisiae* upon red light stimulation. The split transcription factor is built from the plant-derived optical dimer PhyB and PIF3, which is fused to a DNA-binding domain based on the catalytically dead Cas9 protein (dCas9) and a transactivation domain. This design combines at least two major advantages: first, the sgRNAs, guiding dCas9 to the promoter of interest, can be exchanged in an efficient and straightforward Golden Gate-based cloning approach, which allows for rational or randomized combination of up to four sgRNAs in a single expression array. Second, target gene expression can be rapidly upregulated by short red light pulses in a light dose-dependent manner and returned to the native expression level by applying far-red light without interfering with the cell culture. Using the native yeast gene *CYC*1 as an example, we demonstrated that PhiReX 2.0 can upregulate *CYC*1 gene expression by up to 6-fold in a light intensity-dependent and reversible manner using a single sgRNA.

## Introduction

The budding yeast *Saccharomyces cerevisiae* (*S. cerevisiae*) is a popular and well-established host for synthetic biology and biotechnological applications, such as the heterologous production of biofuels, pharmaceuticals, biomaterials, or food supplements.^1−3^ Ideally, these applications employ strains able to grow on sustainable carbon sources.^4^ The implementation of such ambitious processes to develop microbial strains for profitable bioproduction requires extensive metabolic engineering, including a precise adjustment of the expression levels of multiple heterologous and often also of endogenous genes in order to redirect or optimize the metabolic flux through the introduced synthetic pathway—ideally while maintaining the fitness of the strains to ensure sufficient cell growth. Although a huge number of robust and precise molecular tools based on chemical inducers are already available,^5−8^ the necessity of chemical inducers causes limitations such as cell toxicity, non-efficient cellular uptake, interference with host metabolism, and low dynamic reversibility (on/off). To overcome these restrictions, a number of light-inducible regulation systems for application in *S. cerevisiae* came up in the last years using light as an inexpensive and non-toxic inducer, which is compatible with any nutrient composition or carbon source and does not interfere with the host’s metabolism.^9,10^ Figueroa *et al.* and Pérez *et al.* gave a detailed and comprehensive overview of the optogenetic tools available in *S. cerevisiae*, including those for light-regulated gene expression, subcellular protein localization, and protein activity.^11,12^ These reviews show the variety of light-inducible transcription factors (TFs) based on photoreceptors from diverse organisms responding to different wavelengths. However, in most of these systems, the employed transcription factors make use of DNA-binding domains (DBDs) with invariant DNA-binding sites (DBSs). Depending on the system, these sites are either already present in the host genome (*e.g.*, Gal4 binding site^9,13,14^) or have to be engineered into the genome in order to allow transcriptional regulation of the target gene (*e.g.*, EL222^15,16^). Manipulating the transcription level of native genes without engineering the promoter region would require synthetic TFs with tunable binding domains that can be flexibly designed to bind to endogenous promoter sequences. So far, only two light-induced systems based on programmable TFs are available in yeast: The PhiReX 1.0/1.1 systems^17^ previously published by our lab use synthetic transcription activator-like effectors (synTALEs) as DBDs which are programmed to target the *CYC*1 minimal promoter upstream of the gene of interest. Due to the negligibly low basal gene expression levels in an uninduced state and an effective fold induction upon red light induction, these systems are perfectly suited to regulate heterologous genes: PhiReX 1.0 enables 11-fold induction and absolute expression levels similar to the strong *TDH*3 promoter, while PhiReX 1.1 realizes regulation of potentially toxic genes with nearly no background activity and a fold induction of up to 41-fold with expression levels similar to the medium strong yeast *ADH*1 promoter.^17^ However, reprogramming the target site of the synTALE-based TF requires laborious and time-consuming cloning work. The CRISPR/dCas9-based LINuS^18^ system overcomes the limitations associated with synTALE TFs and allows a regulated nuclear import of the TF upon blue light-mediated unfolding of the Jα helix of the second Light Oxygen Voltage (LOV2) domain of *Avena sativa* phototropin 1 (AsLOV2), which releases the nuclear localization signal (NLS) for recognition by importins. After nuclear import, the sgRNA directs the dCas9-Mxi1 fusion protein to the promoter region of the target gene, which is then repressed by the mammalian transcriptional repressor domain Mxi1. This system can be easily directed to different promoter sequences by changing the sgRNA in a simple cloning approach.

Summarizing, the available yeast optogenetics toolbox covers a variety of possibilities to fine-tune the protein levels of heterologous and endogenous genes when those genes are regulated by synthetic promoter sequences. Further, the LINuS system allows moderate repression of endogenous genes under the regulation of their native promoter.^18^ However, the yeast community is still lacking a system that allows reversible overexpression of individual native genes to realize an efficient flux through a certain (heterologous) biosynthetic pathway while the yeast culture can grow under native regulation.

**Figure 1:**
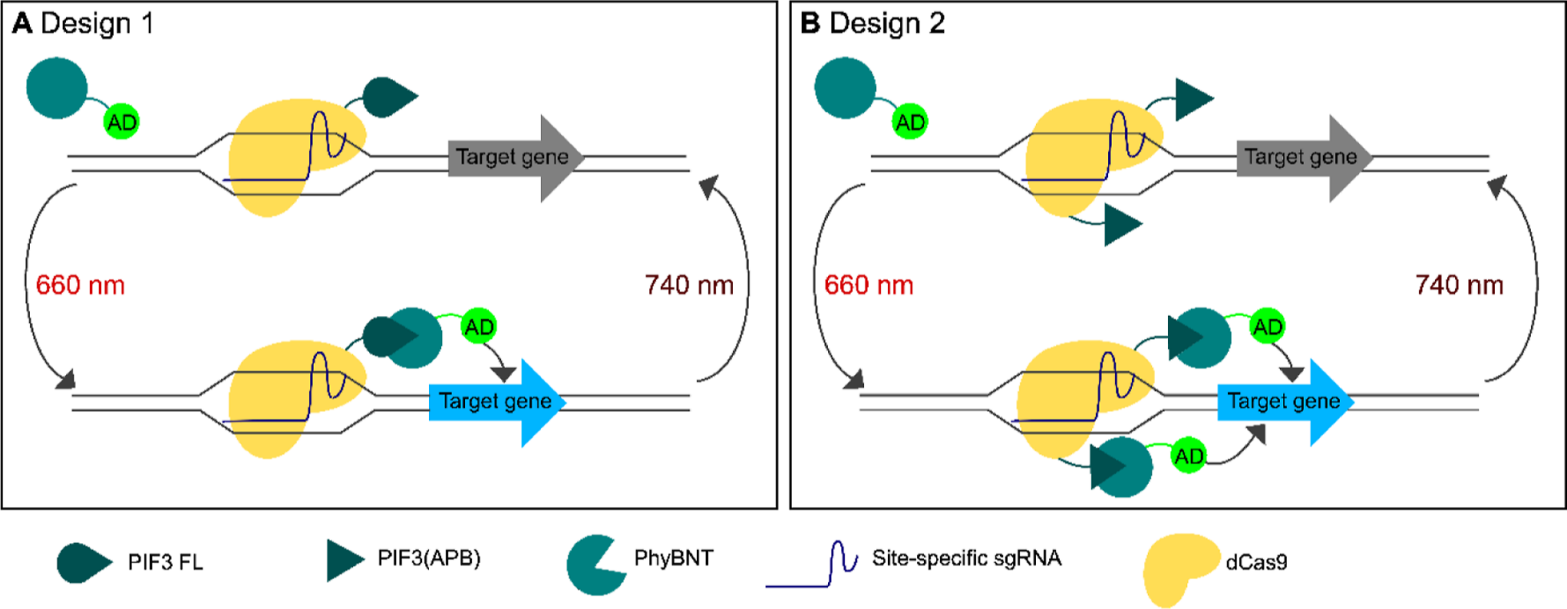
Schematic overview of the red light-inducible dCas9-based TF PhiReX 2.0. (A) Mode of action of design 1. In the dark or in far-red light conditions (λ = 740 nm), the full-length PIF-dCas9 fusion binds to its DBS and the PhyB-AD fusion is located in the nucleus but does not bind to its interacting factor. Stimulation with red light (λ = 660 nm) induces dimerization of the split transcription factor, resulting in the activation of target gene expression. (B) Mode of action of design 2. In the dark or in far-red light conditions (λ = 740 nm), the PIF3(APB)-dCas9-PIF3(APB) fusion protein binds to its DBS, while the PhyB-AD fusion does not bind to its interacting factor, although located in the nucleus. Red light illumination (λ = 660 nm) results in the activation of target gene expression.

With the aim to complete the existing yeast optogenetics toolbox with a system to flexibly upregulate diverse endogenous genes in a reversible manner, we designed the red light-regulated dCas9-based TF PhiReX 2.0. The split TF is built on two fusion proteins, one consisting of the N-terminal truncation of the photoreceptor Phytochrome B (PhyBNT) from *Arabidopsis thaliana* (*A. thaliana*) fused to an NLS and a transactivation domain (AD) and the other consisting of the dCas9 protein, which is fused to a single copy of the full-length PhyB interacting factor PIF3 (Figure 1A) or two copies of its active PhyB binding site (APB), respectively (Figure 1B). By exchanging the DNA target sequence of the sgRNA on the plasmid level through an efficient Golden Gate-based cloning strategy, PhiReX 2.0 is directed to the promoter region of the gene of interest and enhances the transcriptional output upon red light illumination. In this way, PhiReX 2.0 can time and dose dependently fine-tune protein levels, while protein expression can be switched on and off, even multiple times, by applying light of two different wavelengths. The possibility to reversibly induce gene expression in a time-dependent manner allows PhiReX 2.0 to balance bioproduction with the fitness/growth performance of the host cell, which is essential to optimize product yield. Further, dose-dependent regulation of gene expression will help to optimize flux through a pathway by adjusting the endogenous gene expression levels flexibly and in a cloning free manner. The fact that the adjustment of endogenous protein levels has a positive influence on product yield has already been described several times, e.g., for terpene production,^19^ overproduction of triacylglycerols (TAGs),^20,21^ and high-level production of aromatic chemicals.^22^

## Results and Discussion

PhiReX 2.0, the red light-regulated TF presented here, was designed to allow a reversible and time-dependent upregulation of endogenous yeast genes. While our new light switch is based on the same optical dimer from *A. thaliana* as our previously published PhiReX 1.0 and 1.1 systems, it differs in the DBD. The PhiReX 1.0 and 1.1 systems are based on a synTALE DBD, which is programmed to target a synthetic minimal promoter regulating a heterologous gene of interest. In contrast, the dCas9-based PhiReX 2.0 facilitates efficient and reversible regulation of native yeast genes without promoter manipulation at the genomic level. PhiReX 2.0 is based on a genome-integrated split TF and an episomal sgRNA expression cassette. The latter allows the rapid exchange of the DNA-binding part in a single Golden Gate-based cloning step in order to target the dCas9-based TF to any endogenous promoter sequence, facilitating precise manipulation of the transcript level of the regulated gene.

### Design of sgRNA Entry and Multimerization Vectors

**Figure 2:**
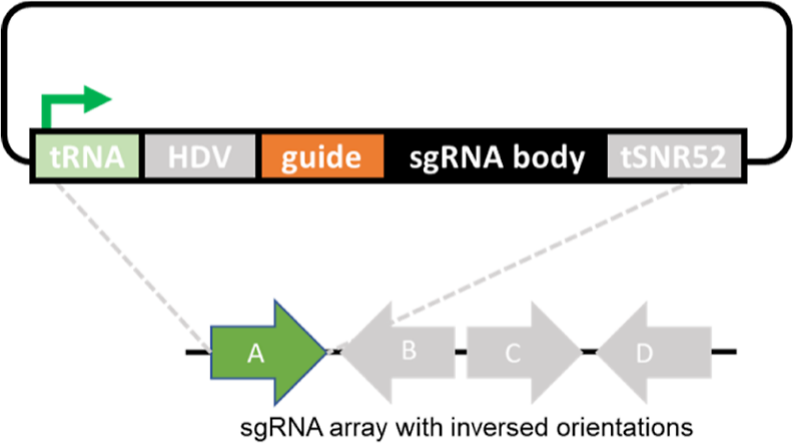
Design of sgRNA arrays. Up to four sgRNAs are encoded on a sgRNA expression plasmid separate from the Cas9/dCas9 expression plasmid, with multiple sgRNA expression cassettes being arranged in alternating orientation. Expression of the sgRNAs is driven by tRNA genes. Upon transcription, the 5′ HDV ribozyme cleaves off the tRNA part, resulting in a functional sgRNA.

The expression of sgRNAs is based on the previously published tRNA-HDV architecture,^23^ which we modified to allow Golden Gate-based cloning of up to four sgRNA cassettes into a single plasmid (see Figure 2 and the Methods section for details). We designed four individual sgRNA cassettes, where the expression of each cassette is driven by a different tRNA, followed by a self-cleaving HDV ribozyme, the 20-bp guide sequence defining the target site, and the chimeric sgRNA scaffold. In addition to the previously used tRNA^Tyr^, tRNA^Pro^ and tRNA^Phe^ promoters, we used the tRNA^Cys^ promoter as the fourth one. A further expansion of our toolkit for arrays containing more than four sgRNAs is possible by implementing additional tRNAs as promoters.

We constructed a set of sgRNA entry vectors containing the described sgRNA cassettes (Table 1). The entry vectors allow a simple and efficient cloning of the 20-bp guide sequences in an oligonucleotide-based manner. The choice of a specific entry vector holding an individual sgRNA cassette defines the sgRNA position within the intended array of multiple sgRNAs, which can be assembled into multi-sgRNA plasmids by Golden Gate cloning. To minimize direct sequence repeats between the identical sgRNA bodies, the Golden Gate adapter sequences were designed in such a way that all sgRNAs within an array are oriented in alternating orientations. This strategy is also intended to reduce transcriptional interference between the individual expression units. These multimerization plasmids were designed to either co-express Cas9/dCas9 or to hold only the sgRNA array (Supporting Information Table S1).

**Table 1:** Overview of Sequence Features in sgRNA Entry and Adapter Vectors^a^

plasmid	left assembly connector	tRNA-HDV-sgRNA cassette (orientation)	right assembly connector
pFM141Cys	L1 CGTCTCa**CTGA**	tRNA^Cys^-HDV-sgRNA (fwd)	R1 **AACG**aGAGACG
pFM142Phe	L2 CGTCTCa**AACG**	tRNA^Phe^-HDV-sgRNA (rev)	R2 **TATG**aGAGACG
pFM143Tyr	L3 CGTCTCa**TATG**	tRNA^Tyr^-HDV-sgRNA (fwd)	R3 **TTCT**aGAGACG
pFM144Pro	L4 CGTCTCa**TTCT**	tRNA^Pro^-HDV-sgRNA (rev)	R4 **ATCC**aGAGACG
pUC19ΔBsmBI_L2	L2 CGTCTCa**AACG**	(n.a.)	Rx **GAGT**aGAGACG
pUC19ΔBsmBI_L3	L3 CGTCTCa**TATG**	(n.a.)	Rx **GAGT**aGAGACG
pUC19ΔBsmBI_L4	L4 CGTCTCa**TATG**	(n.a.)	Rx **GAGT**aGAGACG
pUC19ΔBsmBI_L5	L5 CGTCTCa**ATCC**	(n.a.)	Rx **GAGT**aGAGACG

a Sequences shown in bold are used as Golden Gate adapters. n.a., not applicable.

### Testing the Efficiency and Stability of sgRNA Expression Cassettes

The efficiency of each of our four different sgRNA designs was individually verified using a 20-bp sgRNA sequence targeting the *ADE*2 locus for Cas9-based gene knockout (Supporting Information Figure S1A). We observed the efficient disruption of the *ADE*2 locus, which is easily detected based on the red color of *ade*2 cells–with deletion efficiencies of >95%, which is comparable to the control setup employing the commonly used *SNR*52 promoter to drive sgRNA expression. This indicates that all tRNAs used here result in a sufficient sgRNA transcription.

To asses if the sgRNA arrays we intended to build are stable over longer periods of cultivation, we constructed a 4x sgRNA array and transformed it into yeast BY4742. The resulting transformants were cultivated for ∼100 generations. Subsequent analysis of sgRNA arrays isolated from individual colonies revealed the absence of recombination events. We conclude that arrays with up to four sgRNAs are genetically stable, even if used in longer experiments (Supporting Information Figure S1B).

### Single and Multiple sgRNAs Confer Transcriptional Activation

**Figure 3:**
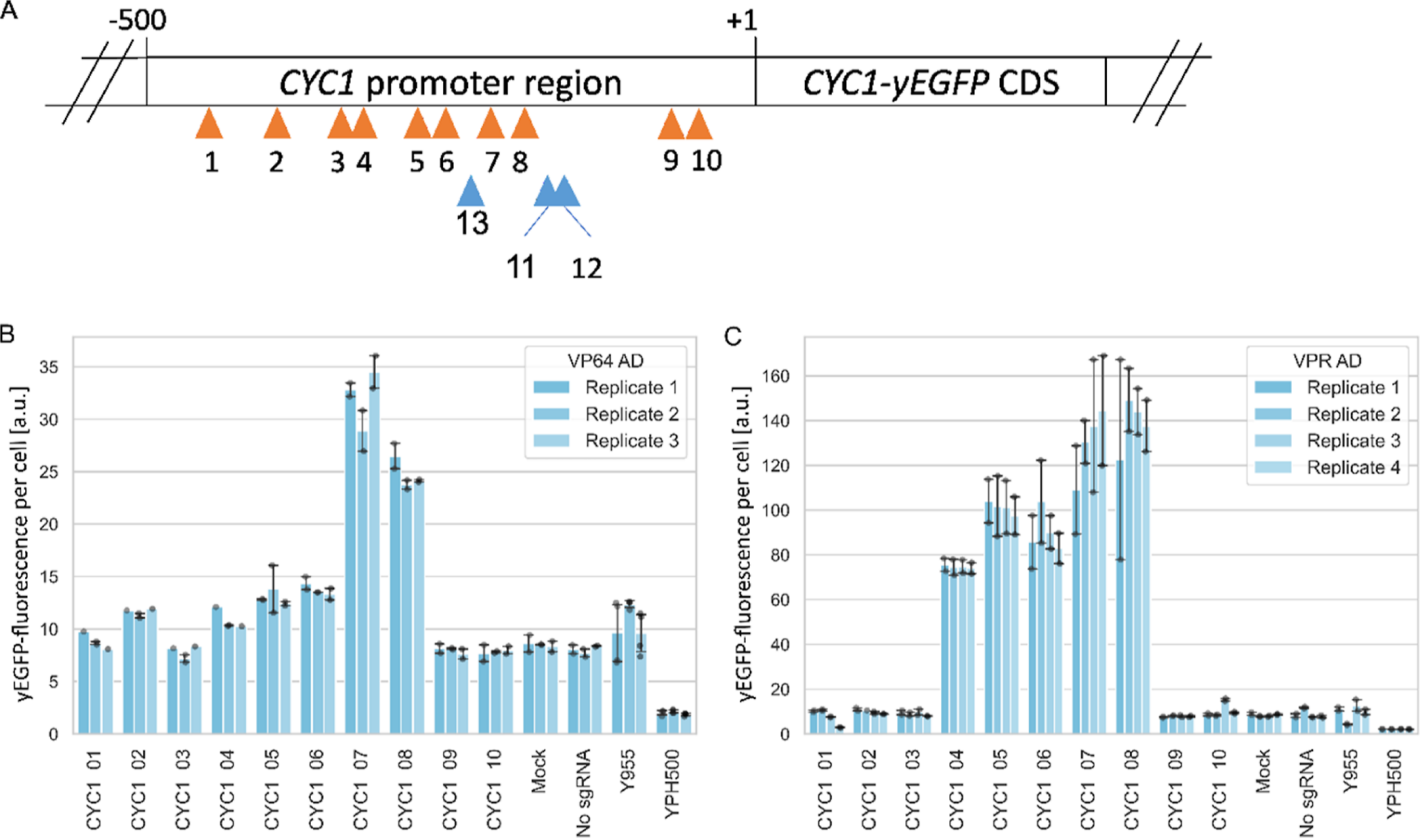
dCas9-mediated transcriptional activation of *CYC*1. (A) Positions of sgRNA targets in the 500-bp region upstream of the *CYC*1-*yEGFP* start codon. Orange arrows indicate the initially designed sgRNAs used for the experiments shown in panels B and C. Blue arrows indicate the additionally designed sgRNAs, which were not measured individually (see the main text for details). (B) yEGFP fluorescence measured in the reporter strain Y955 transformed with dCas9-VP64 and the indicated single sgRNAs. yEGFP output was measured *via* flow cytometry for three biological replicates, each with one or two technical replicates (indicated for each bar as gray dots). (C) yEGFP fluorescence measured in strain Y955 transformed with dCas9-VPR and the indicated single sgRNAs. yEGFP output was measured *via* flow cytometry for four biological replicates, each with two technical replicates (indicated for each bar as gray dots). a.u., arbitrary units.

To test our sgRNA expression system in the context of gene activation, we designed 10 different sgRNAs targeting different positions in the promoter region (500 bp upstream of the start codon) of the *CYC*1 gene (Figure 3A and Supporting Information Table S2), using Benchling [Biology software] (2018). To allow easy assessment of *CYC*1 gene expression, we constructed the reporter strain Y955 by inserting the *yEGFP* CDS as translational fusion to the C-terminus of *CYC*1. All the 10 individual sgRNA constructs were tested in the *CYC*1-*yEGFP* reporter strain Y955, along with a constitutively expressed *dCas*9 fused to either the VP64 or the VPR AD (Figure 3B,C). For both ADs, sgRNAs 7 and 8 resulted in the highest induction of *CYC*1 expression, with VPR mediating a much stronger activation of ∼14-fold and ∼16-fold for sgRNAs 7 and 8, respectively, as compared to VP64 (∼3-fold and ∼2-fold for sgRNAs 7 and 8).

We further tested if a combination of multiple sgRNAs would enhance the transcriptional activation mediated by the VP64 AD. However, since the rational combination of up to three sgRNAs yielded no or only moderate improvements (Supporting Information Figure S2A), we decided to continue with the VPR domain and take a randomized approach to identify a highly activating sgRNA array (Supporting Information Figure S2B). To this end, we designed three additional sgRNAs targeting the *CYC*1 promoter in the region of −150 to −250 bp relative to the translation start site (Figure 3A and Supporting Information Table S2). This region is of special interest since the strongest activation was achieved by sgRNAs 7 and 8, targeting the same area (Figure 3B,C). We randomly cloned all 13 single sgRNAs into each of the sgRNA entry vectors pFM141-143, resulting in three pools of plasmids, each containing all sgRNAs targeting the *CYC*1 promoter. The three pools were then used for sgRNA multimerization in pFM158, resulting in a library of plasmids with random 3x sgRNA arrays, targeting the *CYC*1 promoter. We transformed the reporter strain Y955 with this library and analyzed a pool of the resulting transformants by fluorescence-activated cell sorting (FACS). We used the sorting function to recover cells with the highest yEGFP fluorescence (top 2% of the population). The recovered cells were then grown into individual colonies and reanalyzed for yEGFP fluorescence, and the respective sgRNA array was identified by sequencing (Supporting Information Figure S2). However, the best performing array did not perform better than the single sgRNAs 7 and 8 alone. Interestingly, the best random array does not even contain sgRNA 7 or 8 but consists of a combination of sgRNAs 5, 10, and 11 (Supporting Information Figure S2B). It may be speculated that a synergistic effect for the strongest activating sgRNAs cannot be observed because of, *e.g.*, steric hindrance between individual dCas9 molecules binding to multiple sites in close proximity. Nevertheless, multi-sgRNA arrays might be applied for simultaneous targeting of multiple different promoters. For the implementation of the light-activated regulator, we thus decided to focus on the single sgRNA 8.

### Design of the Red/Far-Red Light-Regulated TFs

**Figure 4:**
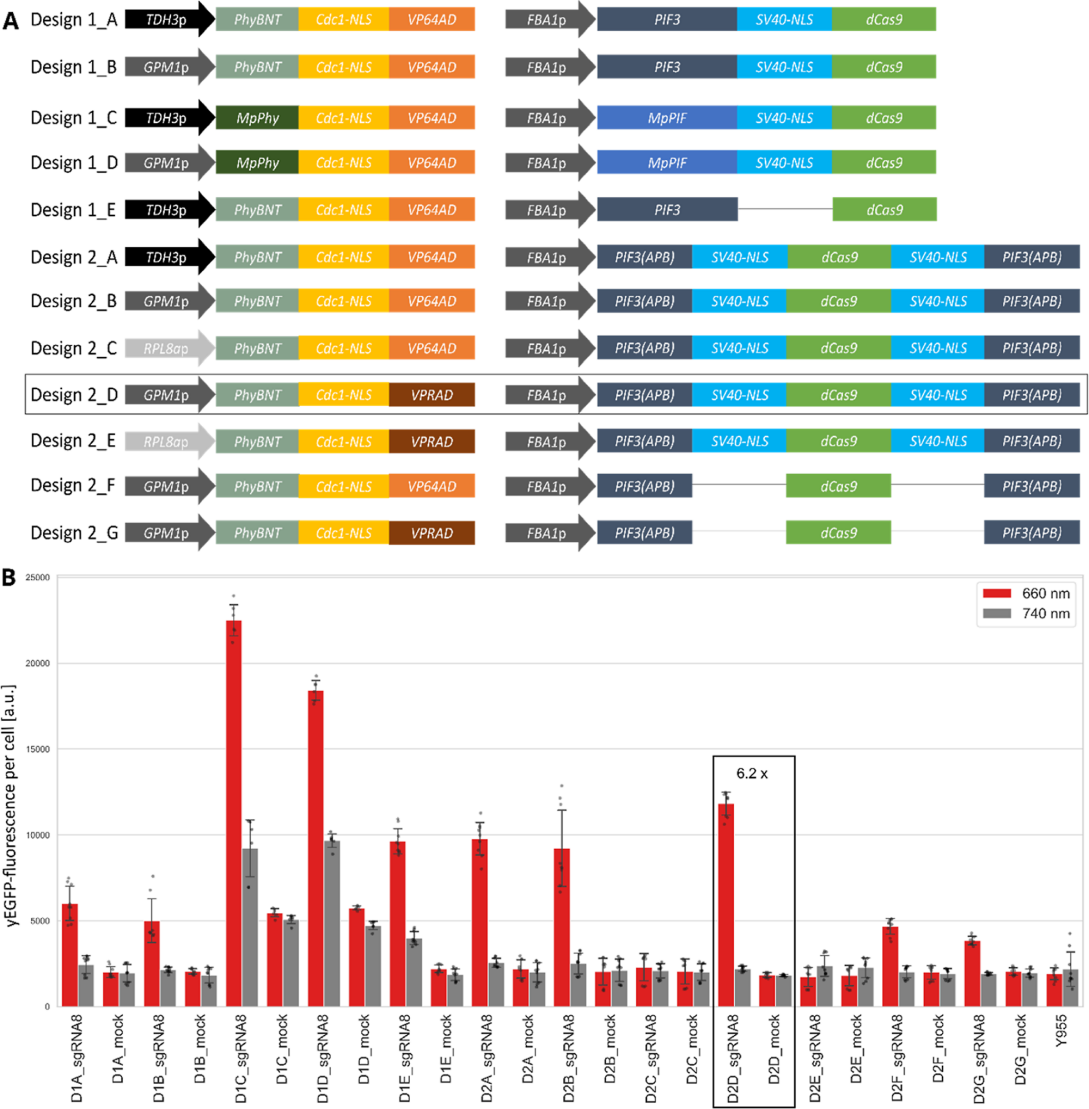
Design and testing of different designs of the light-inducible TFs. (A) Schematic overview of the different designs of both fusion proteins of the split TF. Shown are the key components of each design. (B) PhiReX strains were grown in SD-Ura medium with 25 μM PCB for 6 h in darkness. Induced cells were treated with 10 s red light pulses (660 nm) every 30 min for 16 h. Uninduced cells remained in the dark. Output of yEGFP fluorescence was measured *via* flow cytometry. Each column represents three biological replicates. Technical replicates are indicated as gray dots, error bars indicate standard deviation. a.u., arbitrary units. Design 2_D (dubbed PhiReX 2.0), the best light-regulated TF tested in this study, is highlighted by a black frame in panels A and B, fold induction is indicated.

In order to design a robust light-inducible split TF for the upregulation of endogenous genes with the highest possible activation after red light illumination, and lowest expression in non-inducing conditions, we constructed and tested different variants of the optical dimer (Figure 4A). All constructs share the same general architecture based on two fusion proteins: the photoreceptor fused to an AD and the dCas9 protein fused to the PIF protein. For the construction of the Phy-AD fusion protein, we tested the N-terminal version of Phytochrome B (PhyBNT) from *A. thaliana* and the full-length version of the *Marchantia polymorpha* (*M. polymorpha*) phytochrome (MpPhy) as well as two different ADs (VP64 and VPR). Furthermore, two different designs of the PIF-dCas9 fusion protein were tested: the full-length PIF3 protein from *A. thaliana* or the full-length PIF from *M. polymorpha* (MpPIF) N-terminally fused to an NLS and the dCas9 protein (Figure 1A, design 1) or the dCas9 protein fused to an NLS and the active phytochrome binding domain (APB) of the PIF3 protein (PIF3(APB)) from *A. thaliana* on both the N-terminus and the C-terminus (Figure 1B, design 2). The APB domain was previously shown to be necessary and sufficient for binding to PhyB.^24^ The simultaneous use of two APB motifs allows the recruitment of two PhyB-AD proteins per dCas9 protein. In a blue light-inducible CRISPR/Cas9 system in mammalian cells, it was previously shown that a similar design based on the optical dimers CRY1 and CIB1 yielded much higher mRNA levels after transcriptional activation, compared to CIB1 fusions to only the N- or C-terminus of the dCas9 protein.^25^ Both dCas9-PIF architectures were also tested without NLS to prevent unintended dimerization in the nucleus. Here, nuclear translocation and activation of transcription only occur upon red light-induced dimerization with the NLS-holding Phy-AD protein in the cytosol. Besides the different designs, we compared different promoters driving the transcription levels of Phy-AD and PIF-dCas9 fusion proteins. In contrast to the episomal dCas9-VPR system described above, we here integrated the constructs encoding the optical dimer into the *ura*3-52 locus of the genome. Thereby, we intended to increase genetic stability in the absence of selection markers and to reduce cell-to-cell variability due to variations in plasmid copy number at the cost of possibly lower expression and induction levels.

### Testing Red/Far-Red Light-Regulated TFs for Their Ability to Upregulate the Native *CYC*1 Gene

To test the designed constructs for their ability to reliably activate the constitutive *CYC*1 gene, we co-expressed either sgRNA 8 targeting the *CYC*1 promoter, which regulates the *CYC*1-*yEGFP* fusion protein (reporter strain Y955), or a mock sgRNA, which does not bind in the yeast genome, along with the different genome-integrated red light-regulated split TFs. Both combinations were then tested for their yEGFP fluorescence in an uninduced (single far-red light pulse, then dark) or induced (red light treatment) state *via* flow cytometry (Figure 4B and Supporting Information Figure S3A,B). All constructs based on the *A. thaliana* PhyBNT and PIF3 interaction resulted in low background levels of the reporter fluorescence when tested with the mock sgRNA in the induced or uninduced state. On the contrary, constructs based on the optical dimer from the liverwort *M. polymorpha* exhibited much higher background levels with the mock sgRNA and even higher reporter expression in the uninduced state with sgRNA 8 (Figure 4A and B; design 1_C and D). Due to this high background expression, we decided to continue our experiments with the optical dimer from *A. thaliana*, although the TF based on the optical dimer from *M. polymorpha* resulted in the highest absolute *yEGFP* expression values after light induction (design 1_C). Since the ratio of the two TF components (DBD- and AD-fusion proteins) is considered to be crucial for reliable and specific dimerization,^26^ the light-regulated TFs of design 1_A and B differ in the expression strength of the PhyBNT-NLS-AD fusion protein due to employing either the very strong *TDH*3 (design 1_A) or the strong *GPM*1 promoter (design 1_B). With reference to a previous characterization of these promoters regulating *yEGFP*,^27^ we expected protein ratios of the PhyBNT-NLS-AD fusion and the PIF3-NLS-dCas9 or PIF3(APB)-NLS-dCas9-NLS-PIF3(APB) fusion proteins regulated by the strong endogenous *FBA*1 promoter of 2:1 for design A and 1:1 for design B. However, in the study presented here, the ratio of PhyB and PIF3 does not seem to have a crucial impact on the performance of the split TF since both TFs revealed comparable results with a moderate fold induction of approximately three after light induction and low expression levels in uninduced conditions. To reduce the background fluorescence even further, we deleted the NLS in the PIF3-dCas9 fusion protein (design 1_E) to prevent dCas9 from entering the nucleus prior to light induction. Against our expectation, design 1_E resulted in background levels that were even higher than before for design 1_A.

A clear improvement in fold change induction was gained by changing design 1, which is based on the full-length Arabidopsis PIF3 protein, to design 2 where two PIF3(APB) truncations were N- and C-terminally fused to the dCas9 protein. Design 2 results in 5 times higher expression after light induction with VP64 AD (design 2_A and B) and six-fold induction with the VPR AD (design 2_D). Similar to the results obtained above for design 1, the ratio of the two parts of the optical dimer is also not crucial for design 2 (design 2_A and B). Overall, the results suggest that higher expression levels of the PhyBNT-NLS-AD fusion protein are required for a good performance of the TF, as constructs regulated by the weak yeast promoter *RPL*8*a* show inefficiently low induction rates (design 2_C and E). In contrast to our results for designs 1_A–E, the NLS deletion in the PIF3(APB)-dCas9-PIF3(APB) protein (design 2_F and G) showed the desired off-state, at the cost of a considerably lower induction rate of around 2 compared to design 2_B and D.

Based on the findings described above, design 2_D provides the optimal combination of low background activity in the off-state, and a strong ∼6-fold induction of the *CYC*1-*yEGFP* gene after red light illumination. The corresponding construct was named PhiReX 2.0. It should be noted that the fold induction achieved with PhiReX 2.0 is highly dependent on the native expression level of the target gene and, therefore, high fold changes—as previously achieved with PhiReX 1.1 for the regulation of heterologous genes with neglectable expression levels in non-induced state—cannot necessarily be expected for the upregulation of endogenous genes.

### Characterization of PhiReX 2.0

**Figure 5:**
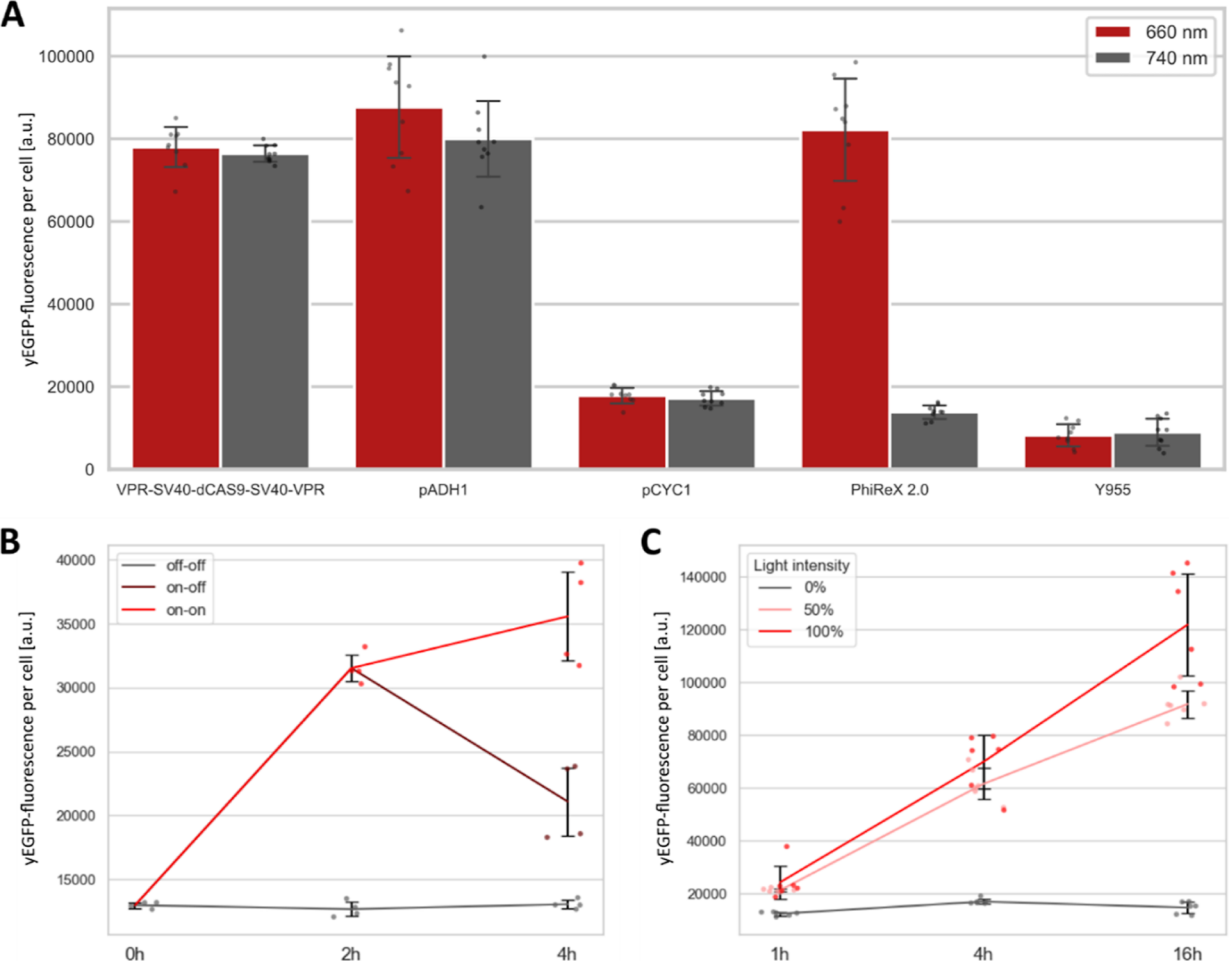
Characterization of PhiReX 2.0. Yeast cells were grown in a 24-well plate in SD-Ura-Leu medium with 25 μM PCB for 6 h in darkness prior to induction. *yEGFP* gene expression was measured *via* flow cytometry, and values are given as the geometrical mean of yEGFP-fluorescence per cell. (A) PhiReX 2.0 in comparison to VPR-SV40-dCas9-SV40-VPR (a constitutively expressed dCas9 protein, which is N- and C-terminally fused to the SV40 NLS and the VPR AD), pADH1 (*yEGFP* under regulation of the native *ADH*1 promoter), and p*CYC*1 (*yEGFP* under the regulation of the native *CYC*1 promoter). Uninduced cells were grown in the dark for 16 h, while induced cells were treated with a single 30 s red light pulse, followed by 10 s pulses every 30 min. Each column represents three biological replicates, with three technical replicates each (indicated as gray dots), error bars indicate standard deviation. (B) Reversibility of protein production *via* PhiReX 2.0. Uninduced cells were grown in the dark for 4 h after an initial far-red light pulse. Induced cells were treated with a single 30 s red light pulse, followed by 10 s pulses every 30 min for 4 h. Reversion of gene expression was mediated by a 30 s far-red light pulse after culturing for 2 h in inducing conditions. After far-red light treatment, cultures were grown for 2 h in the dark. yEGFP output was measured *via* flow cytometry for two biological replicates, each with two technical replicates. Single values are indicated by dots, and error bars indicate standard deviation, a.u.: arbitrary units. (C) Light dose dependency of PhiReX 2.0. Uninduced cells were cultured in the dark for 16 h. Induced samples were activated with a single light pulse of the indicated light intensity (100% ≙ 28 W/m^2^ and 50% ≙ 14 W/m^2^). Post-induction, cells were grown for 16 h with 10 s pulses every 30 min of the same light intensity. yEGFP-fluorescence was measured *via* flow cytometry for three biological replicates, each in duplicate. Single values are represented as dots, and error bars indicate standard deviation, a.u.: arbitrary units.

To investigate the induction efficiency of PhiReX 2.0, expression levels in induced and non-induced conditions achieved by the light-inducible TF were compared with native constitutive yeast promoters and a constitutively expressed dCas9, which is N- and C-terminally fused to a SV40 NLS and a VPR AD. When PhiReX 2.0 is targeted to the promoter region of the native *CYC*1 gene in the reporter strain Y955, PhiReX 2.0 achieves a fold induction of ∼6, reaching fluorescence levels of the medium strong native *ADH*1 promoter after a growth period of 16 h in inducing conditions (Figure 5A). In non-inducing conditions, *yEGFP* expression levels are comparable to *yEGFP* expression regulated by the native *CYC*1 promoter as expected. Further, upon red light treatment, PhiReX 2.0 achieves strong yEGFP fluorescence comparable to that obtained with constitutively expressed dCas9. This indicates that dimerization of the two parts of the split TF after red light induction is highly efficient. To provide potential users of PhiReX 2.0 with a full characterization of the system, we also demonstrated reversibility of gene expression activation and light dose dependency (Figure 5B and C). To test the reversibility of PhiReX 2.0, we cultured yeast strains for 6 h in the dark before starting three different culture conditions: “off–off”-samples were kept for 4 h in the dark. “On–on”-samples were induced with a single 30 s red light pulse and cultured for 4 h with 10 s pulses given every 30 s, “on–off”-samples were induced as described for “on–on”-samples and grown for 2 h with red light pulses; thereafter, induction was reversed by a 30 s far-red light pulse. Figure 5B clearly shows the downregulation of *yEGFP* expression after far-red light treatment. Moreover, we observed that the level of gene expression can be regulated by the applied light intensity. To this end, we cultured PhiReX 2.0 cells for 16 h in the dark or with red light pulses of 50 and 100% light intensity after an initial 6 h growth phase in the dark. While the cultures treated with light pulses of 100% intensity for 16 h showed a fold induction of 8.3, cultures grown with pulses of 50% light intensity showed a fold induction of 6.3. Furthermore, to investigate if the expression of the split TF in strain PhiReX 2.0 affects growth, we cultured control strain Y955 (harboring the *CYC*1-*yEGFP* reporter) and PhiReX 2.0 in 96 well microtiter plates, starting from an inoculation OD_600_ of 0.01 (Supporting Information Figure S4A). Based on the recorded growth curves, both strains show virtually identical growth characteristics with doubling times of 2.6 h (Y955, μ = 0.27 h^–1^) and 2.5 h (PhiReX 2.0, μ = 0.28 h^–1^) and lag times of 10.1 and 8.2 h, respectively. We further investigated if red light induction negatively affects the growth. For this, samples were grown in 24-well plates and either kept in darkness after an initial far-red light treatment (uninduced samples; “–“) or subjected to red light induction after an initial pre-incubation in darkness for 6 h (induced samples; “+”) (Supporting Information Figure S4B**)**. In this setup, the final OD_600_ was slightly lower for PhiReX 2.0 (OD_600_[PhiReX 2.0-] = 2.7 and OD_600_[PhiReX 2.0+] = 2.9) than the control strain (OD_600_[Y955-] = 3.9 and OD_600_[Y955+] = 3.7). However, induction with red light did not affect the growth of either strain. Thus, in conclusion, the PhiReX 2.0 strain has very good potential for bioproduction purposes.

### Cellular Localization of the Optical Dimer

To investigate if insufficient nuclear import might cause the low induction observed for design 2_F and 2_G, we replaced the AD and the dCas9 protein with the fluorescence reporters mScarlet and yEGFP, respectively, and analyzed the subcellular localization of the newly designed constructs *via* confocal microscopy (Supporting Information Figure S5). DAPI staining was performed to visualize the nucleus. The yeast strain with design 2_yEGFP, which carries only the PIF3(APB)-yEGFP-PIF3(APB) protein (lacking the PhyBNT-NLS-mScarlet protein), shows, as expected, a clear localization of the yEGFP fusion protein in the cytoplasm. In strain design 2_mScarlet_yEGFP, containing both proteins of the optical dimer, PIF3(APB)-yEGFP-PIF3(APB) is partly available in the nucleus in uninduced conditions, while most of the protein is still located in the cytoplasm. In the same strain, the PhyBNT-NLS-mScarlet fusion protein containing an NLS is located in the nucleus. After light induction, a clear shift of the PIF3(APB)-yEGFP-PIF3(APB) protein becomes visible, indicating that the nuclear transport of the dimerized proteins is functional. Although this design was not beneficial for the activating system established here, it will be a good starting point for the establishment of a light-inducible transcriptional repressor or a Cas9 nuclease enabling light-regulated genome editing.

## Conclusions

With numerous designs tested in this study, we identified design 2_D to be the superior optogenetic tool and named it PhiReX 2.0. PhiReX 2.0 is built from an optical dimer established on the basis of *A. thaliana* proteins in combination with the dCas9 protein and the VPR AD. The PIF3(APB)-dCas9-PIF3(APB) architecture allows for targeting of two ADs to the promoter region, thereby increasing the expression levels after light induction. In contrast to this approach, targeting the *CYC*1 promoter with multiple sgRNAs remained less effective compared to a single sgRNA utilization.

The PhiReX 2.0 TF presented here mediates a fold induction of 6.2 after 16 h of growth in red light when targeting the native *CYC*1 promoter, while leaky expression in non-induced conditions is negligibly low. Further, PhiReX 2.0 can control the gene expression of endogenous yeast genes in a reversible and light-dose-dependent manner. The expression of the genome-integrated optical dimer has only slight effects on growth performance and biomass accumulation.

## Methods

### Strains and Cell Culture

*Escherichia coli* (*E. coli*) strains DH5α, NEB5α, and NEB10β (New England Biolabs, Frankfurt am Main, Germany) were used. Cells were grown at 37 °C and 230 rpm in Luria-Bertani (LB) medium, with the appropriate selection marker (kanamycin or ampicillin with a final concentration of 50 mg/mL). For yeast experiments, *S. cerevisiae* strain YPH500 (ATCC 76626) was cultured at 30 °C and 200 rpm in Yeast extract Peptone Dextrose Adenine (YPDA)-rich medium or in appropriate Synthetic Dextrose (SD) medium to allow for selection of transformed cells.

### DNA Assembly Methods and Cell Transformation Protocols

*In vitro* overlap-based cloning was done using the NEBuilder HiFi DNA Assembly Master Mix (New England Biolabs, Frankfurt am Main, Germany) according to the manufacturer’s recommendations or the SLiCE method.^28,29^*E. coli* transformations were performed according to the high-efficiency transformation protocol from New England Biolabs.

For *in vivo* DNA assembly, *S. cerevisiae* transformations were carried out following the LiAc/SS carrier DNA/PEG method by Gietz and Schiestl.^30^ The correctness of DNA assemblies was verified by sequence analysis (LGC Genomics, Berlin, Germany).

Multi-gene constructs were assembled using the AssemblX toolkit,^27,31^ which enables level-based multi-part assemblies. While Level 0 plasmids are generally employed to assemble single transcriptional units (TUs), Level 1 vectors allow the combination of up to five Level 0 modules. Sequence information of all constructs described here are given in Supporting Information File 1. Sequences of oligonucleotides are available in Supporting Information Table S3.

PCR amplifications were done using either PrimeSTAR GXL DNA polymerase (Takara Bio, Saint-Germain-en-Laye, France) or Phusion polymerase (Life Technologies, Darmstadt, Germany) according to the manufacturer’s instructions. Restriction enzymes were purchased from New England Biolabs and used following the manufacturer’s protocols.

### Cloning of sgRNA Entry and Multimerization Vectors

We designed a Golden Gate-based strategy, which allows the *Btg*ZI-mediated cloning of 20-bp sgRNA guide sequences into specific sgRNA entry vectors. Subsequently, sgRNA cassettes can be assembled into multi-sgRNA arrays, employing a *Bsm*BI-mediated Golden Gate reaction.

The sgRNA entry vectors used in the present study are based on pUC19 (New England Biolabs) and follow the tRNA-HDV architecture^23^ with some modifications, detailed above (Figure 2). This sgRNA expression strategy utilizes tRNA genes, which are transcribed by RNA Polymerase III and serve as promoter for sgRNA expression. The resulting transcript contains the full tRNA, followed by the hepatitis delta virus (HDV) ribozyme and the sgRNA. Upon transcription, the HDV ribozyme cleaves off the tRNA part, resulting in a functional sgRNA transcriptionally fused to a 5′ HDV ribozyme. For the construction of sgRNA entry vectors, pUC19 was modified using a combination of SLiCE^28,29^ and NEBuilder HiFi DNA Assembly. In short, the *Bsm*BI site was deleted from pUC19 to render the plasmid compatible to our Golden Gate cloning strategy. The resulting pUC19Δ*Bsm*BI was further modified by insertion of a sgRNA expression cassette, driven by the commonly used *SNR*52 promoter, a *URA*3 marker gene, and a 2μ origin of replication. The resulting plasmid pFM141(SNR52) was modified by replacing the *SNR*52 sgRNA expression cassette with one of four different tRNA-HDV combinations, along with a *Btg*ZI-flanked *ccdB* cassette (serving as the cloning site for 20-bp sgRNA guide sequences) and the sgRNA scaffold. All parts were assembled into pFM141(SNR52) by NEBuilder HiFi DNA Assembly to yield vectors pFM141Cys and pFM142-pFM144, each with a specific combination of L and R assembly connectors (necessary to allow for sgRNA multimerizations) flanking each single sgRNA assembly cassette. Each connector of type R is compatible with the L connector in the next sgRNA entry vector to allow the assembly of predefined sgRNA arrays (*e.g.*, R1 is compatible with L2 and R2 with L3) (Table 1).

To allow assembly of multiple sgRNAs into a single array, the multi-sgRNA expression plasmids pFM149-pFM153 were designed based on the previously published AssemblX vector series pL0_A0_1; pL0_B0_1; pL0_C0_1; pL0_D0_1; and pL0_E0_1.^27,31^ To achieve compatibility with the Golden Gate strategy for sgRNA multimerization, we deleted the *Bsm*BI sites in these vectors. A *ccdB* selection cassette flanked by assembly connectors L1 and Rx was inserted between the AssemblX homology regions of each vector to yield plasmids pFM0149-pFM0153. While the L1 connector is compatible with the L1 connector in pFM141Cys, the Rx connector is not compatible with any of the R connectors in pFM141Cys-144; in this way, our system allows the facile generation of sgRNA arrays with less, or even more than four sgRNAs. Thus, to enable assembly of arrays containing one to three sgRNAs, we designed assembly adapters containing assembly connector Rx in combination with adapters L2–L5 (Table 1). To this aim, we used R2O designer^32^ to generate a 320 bp biologically neutral linker sequence flanked by L2–L5, each in combination with Rx. The linker fragment was subcloned into pUC19Δ*Bsm*B to yield pUC19Δ*Bsm*B_L2–L5. Cloning of arrays with more than four sgRNAs requires the design of additional sgRNA expression cassettes with compatible adapter sequences.

### Cloning of Cas9 and dCas9 Expression Plasmids

In addition to the possibility to assemble sgRNA arrays in sgRNA expression vectors, we created different versions of *Streptococcus pyogenes* Cas9 and dCas9 expression plasmids that enable co-expression of Cas9/dCas9 and sgRNAs from a single plasmid (pFM138 and pFM154-159; Supporting Information Table S1). Cas9 was amplified from p414-TEF1p-Cas9-*CYC*1t^33^ (gift from George Church, Addgene #43802), and dCas9 was amplified from pMLM3705^34^ (gift from Keith Joung, Addgene #47754). Generally, the plasmids are based on a modified AssemblX pL1E_hc backbone, containing the pBR322 origin of replication and a kanamycin selection marker for plasmid maintenance in *E. coli*. A 2μ-origin along with a *TRP*1 auxotrophic marker and a dominant selection cassette (hygromycin or G418 resistance) enables maintenance in yeast. A *ccdB* expression cassette, flanked by adapters L1 and Rx for Golden Gate cloning, enables efficient insertion of multiple sgRNA cassettes (see below for the cloning procedure). The dCas9 expression cassette contains a translational fusion of dCas9 and an AD (VP64 or VPR), which is controlled by the *TEF*1 promoter.

### Cloning and Multimerization of sgRNAs

sgRNA guide sequences were ordered as single-stranded oligonucleotides. The upper-strand sequences were ordered as 5′-GATC(N)_20_-3′ and the lower strand sequences as 5′-AAAC(N)_20_-3′, where (N)_20_ represents the intended guide sequence in forward orientation or reverse complementary orientation. Upper- and lower-strand oligonucleotides were mixed equimolarly to a final concentration of 33 μM for each oligonucleotide in 1x CutSmart buffer (NEB). In a PCR cycler, the mixture was heated to 95 °C for 5 min and then slowly cooled to 20 °C in 0.5 °C steps (10 s per step). Annealed oligonucleotides were then diluted 1:200 and cloned into the appropriate sgRNA entry vector, which determines the position of the respective sgRNA cassette in the intended final multi-sgRNA construct. The Golden Gate-based sgRNA cloning reaction setup is as follows: 1 μL of annealed oligonucleotides (1:200 dilution), 80 ng of sgRNA entry plasmid, 1 mM ATP, 1 μL of *Btg*ZI (NEB), and 1 μL of T4 DNA ligase in 1x CutSmart buffer with 20 μL final volume. The assembly reaction was done in a PCR cycler: 25x (2 min at 37 °C and 2 min at 16 °C), followed by 10 min at 60 °C and 20 min at 80 °C. 2–5 μL of the assembly reaction was transformed into chemically competent *E. coli* cells which were selected with carbenicillin. The resulting sgRNA cassettes were sequence-verified and used for multimerization into arrays. To this end, 40 fmol (146 ng) of each sgRNA plasmid to be represented in the intended array was mixed with 20 fmol (68 ng) of the intended target plasmid. It is mandatory that the used sgRNA entry vectors have Golden Gate adapters compatible to each other. Additionally, 40 fmol of the appropriate linker plasmid pUC19Δ*Bsm*BI_Lx is required (*e.g.*, linker plasmid L5 for an assembly with pFM141-144). The assembly reaction was then performed in a final volume of 20 μL, containing the plasmid mix described above in 1x T4 DNA Ligase buffer (NEB), 1 μL of *Esp*3I (NEB), and 1 μL of T4 DNA Ligase (NEB). The assembly reaction was done in a PCR thermal cycler: 30x (5 min at 37 °C, 5 min at 16 °C), followed by 10 min at 37 °C for the final digestion and 20 min at 65 °C for enzyme inactivation. 5 μL of the assembly reaction were transformed into *E. coli* NEB10β cells and selected in LB medium with carbenicillin. Individual clones were analyzed by restriction digestion and sequencing.

### Construction of the Red Light-Inducible dCas9-based TF

The plasmids with the different variations of the dCas9-based red light-inducible TFs (Figure 4A) were assembled using the AssemblX toolkit.^27,31^ The final Level 1 constructs (given in Supporting Information Table S4) were built from preassembled Level 0 plasmids by *in vivo* recombination in yeast.^30^ Level 0 plasmids were assembled using NEBuilder HiFi DNA Assembly Master Mix. Templates and primers for the amplification of DNA fragments needed for the assembly of Level 0 vectors are given in Supporting Information Table S5. Final Level 1 constructs were linearized with *Pme*I and integrated into the *ura*3-52 locus *via* homology regions. Positive cells were selected on the SD-Leu medium and screened *via* colony PCR. The resulting yeast strains were transformed with sgRNA expression plasmids (sgRNA8 or mock), and positive cells were selected on SD-Ura medium and screened *via* colony PCR. Information about the resulting yeast strains is provided in Supporting Information Table S6.

### Cloning of the Fluorescence-Labeled Optical Dimer for Localization Studies

Level 0 constructs pLH_201-203 and Level 1 construct pLH_204 were cloned with the AssemblX toolkit.^27,31^ Templates and primers for PCR amplification of Level 0 parts are given in Supporting Information Table S5. Level 0 plasmids pLH_201 and pLH_202 were used to assemble Level 1 construct pLH_204 (Supporting Information Table S4). Level 1 constructs were linearized with *Pme*I and integrated into the *ura*3-52 locus *via* homology regions. Positive cells were selected on SD-Leu medium and screened *via* colony PCR. Information about the resulting yeast strains is provided in Supporting Information Table S6.

### Induction Experiments

For light induction experiments, cells were inoculated in 500 μL of SD-Ura-Leu medium in 48-well deep-well plates and incubated shaking for 24 h (30 °C, 240 rpm). A main culture was then inoculated in 500 μL of fresh SD-Ura-Leu medium containing 25 μM PCB (Phycocyanobilin, SiChem GMBH, Bremen, Germany) to an OD_600_ ≈ 0.1 and incubated for another 16 h in 24-well plates with a transparent bottom (product no. 303008, Porvair Science Ltd, Norfolk, UK) covered with sterile aluminum sealing foil. Twenty-four-well microtiter plates were placed into a custom-made light plate apparatus device (LPA).^35^ The LPAs were equipped with a 660 nm LED (product no. L2-0-R5TH50-1, LEDsupply, Randolph, VT, USA) and a 740 nm LED (product no. MTE1074N1-R, Marktech Optoelectronics Inc., Latham, NY, USA). For light-sensitive experiments, cultures were treated with a 30 s far-red light pulse and subsequently grown for 6 h at 30 °C and 200 rpm in the dark. Thereafter, induced cells were irradiated with a single 30 s red light pulse, followed by 10 s red light pulses every 30 min over the indicated period, while uninduced cells were cultured in the dark. If not specified otherwise, the light intensity of the 660 nm LED was set to 28 W/m^2^ and that of the 740 nm LED to 69 W/m^2^. All light-sensitive manipulations were done under green safelight.

For experiments with constitutively expressed dCas9-AD constructs, the strains harboring the combined dCas9/sgRNA expression plasmid were pre-cultured in YPDA medium with the appropriate antibiotic (hygromycin or G418) under the same conditions as described above for light induction experiments. The main culture was inoculated to OD_600_ 0.1 in 48-well deep-well plates and grown for another 16 h in YPDA with antibiotic selection prior to FACS analysis.

### Fluorescence-Activated Cell Sorting

After illumination and cultivation, protein production was stopped by adding 500 μg/mL cycloheximide, and cells were filtered through a 40 μm cell strainer (pluriSelect, Leipzig, Germany). The fluorescence output of single cells was measured with a BioRad S3e Cell Sorter with 488 and 561 nm 100 mW lasers (BioRad Laboratories, Munich, Germany). Yeast cells were identified and gated in a forward/sideward scatter dot plot. Within this gate, 20,000 cells were counted per measurement. Fluorescence was analyzed in a histogram, and the geometrical mean fluorescence per cell was calculated for each measurement using the Flowlogic software (Miltenyi Biotec B.V. & Co. KG, Bergisch Gladbach, Germany). All results are shown as the geometric mean calculated from three biologically independent experiments, each with three technical replicates if not stated otherwise. Error bars indicate standard deviation. The fold induction was calculated by normalization to the reporter strain Y955 as control.

### Growth Assays in 96-Well Plates

Growth assays in microtiter plate readers were essentially performed as described.^36^ In short, cells were precultured in an appropriate medium until they reached the late exponential phase. The cells were then washed and diluted in a fresh medium to a starting OD_600_ of 0.01. A 96-well plate was loaded with 200 μL of culture per well and covered with 20 μL of mineral oil to prevent evaporation. Incubation and OD readings were done in a TECAN Infinite 200 PRO instrument at 30 °C with alternating linear and orbital shaking (6 mm amplitude). The OD was recorded every ∼35 min. The resulting growth curves were processed with a custom MatLab script, available on request. Plate reader OD values were calibrated to a standard cuvette OD using a predetermined calibration factor.

### Confocal Microscopy

Fluorescence imaging was performed using a Zeiss LSM880 confocal laser scanning microscope (Zeiss, Jena, Germany) subsequently to the light induction of the yeast cultures. Ten μL of the cell suspension was analyzed with the Plan-Apochromat 63x/1.4 oil DIC M27 objective. For excitation of DAPI (4′,6-diamidino-2-phenylindole), a 405 nm diode laser, for yEGFP, a 488 nm argon laser, and for mScarlet, a 561 nm diode pumped solid-state laser were used with the appropriate main beam splitters. The laser attenuation adjustment was set according to the sample but never exceeded 2% for any wavelength. A gallium arsenide phosphide detector (GaAsP) and a photomultiplier tube (PMT) were used in a double-track mode. A transmitted light image was recorded simultaneously using the transmitted light detector (T-PMT). The detection bandwidths were set to approximately 60 nm width, centered around the emission maxima of the respective fluorophores. The pinhole was set to 1 airy unit. Three times averaging (line mode) with pixel dwell times between 2 and 5 μs was applied.

## Supporting Information Available

Evaluation of the efficient expression and stable propagation of sgRNA cassettes, transactivation of *CYC*1-*yEGFP* expression mediated by rationally or randomly combined sgRNA arrays, characterization of different designs of the light-inducible TF, growth curve of PhiReX 2.0 compared to control strain Y955, confocal microscopy analysis of the cellular localization of the optical dimer, features of sgRNA multimerization vectors, sequences of sgRNA target sites for *CYC*1 with respective percentages of specificity and efficiency (on target) scores, list of oligonucleotides used for cloning, overview of level 0 fragments and backbones used for the assembly of level 1 plasmids, overview of the PCR templates and PCR primers used for the assembly of Level 0 plasmids, and list of yeast strains generated in this study (PDF)
All plasmid maps are available in GenBank format (ZIP)
