# Supplementary material for: PhiReX 2.0: A Programmable and Red Light-Regulated CRISPR-dCas9 System for the Activation of Endogenous Genes in Saccharomyces cerevisiae
Source: ACS Synth Biol. 2023 Apr 4;12(4):1046–57. doi: 10.1021/acssynbio.2c00517 (PMC10127447; doi:10.1021/acssynbio.2c00517)
Supplement: Supplementary file 1 — sb2c00517_si_001.pdf [file sb2c00517_si_001.pdf]

PhiReX 2.0: A programmable and red light-regulated CRISPR-dCas9 system for the activation of endogenous genes in *Saccharomyces cerevisiae*

Fabian Machens[1¶], Guangyao Ran[1¶], Ciaran Ruehmkorff[1], Julie Meyer auf der Heyde[1], Bernd Mueller-Roeber[1,2,3*] and Lena Hochrein[1*]

[1] University of Potsdam, Department of Molecular Biology, Potsdam, 14476, Germany
[2] Max Planck Institute of Molecular Plant Physiology, Potsdam, 14476, Germany
[3] Center of Plant Systems Biology and Biotechnology (CPSBB), Plovdiv, 4000, Bulgaria

[¶] Authors contributed equally to the results presented.
* Corresponding authors
Email:
lena.hochrein@uni-potsdam.de (LH)
bmr@uni-potsdam.de (BMR)

SUPPORTING INFORMATION

Supplementary Figure S1: Evaluation of the efficient expression and stable propagation of sgRNA cassettes.
Supplementary Figure S2: Transactivation of *CYC1-yEGFP* expression mediated by rationally or randomly combined sgRNA arrays.
Supplementary Figure S3: Characterization of different designs of the light-inducible TF.
Supplementary Figure S4: Growth curve of PhiReX 2.0 compared to control strain Y955.
Supplementary Figure S5: Confocal microscopy analysis of the cellular localization of the optical dimer.

Supplementary Table S1: Features of sgRNA multimerization vectors.
Supplementary Table S2: Sequences of sgRNA target sites for CYC1, ALG9, IPP1 and UBC6 with respective percentages of specificity and efficiency (on target) scores.
Supplementary Table S3: List of oligonucleotides used for cloning.
Supplementary Table S4: Overview of Level 0 fragments and backbones used for the assembly of Level 1 plasmids.
Supplementary Table S5: Overview of the PCR templates and PCR primers used for the assembly of Level 0 plasmids.
Supplementary Table S6: List of yeast strains generated in this study.

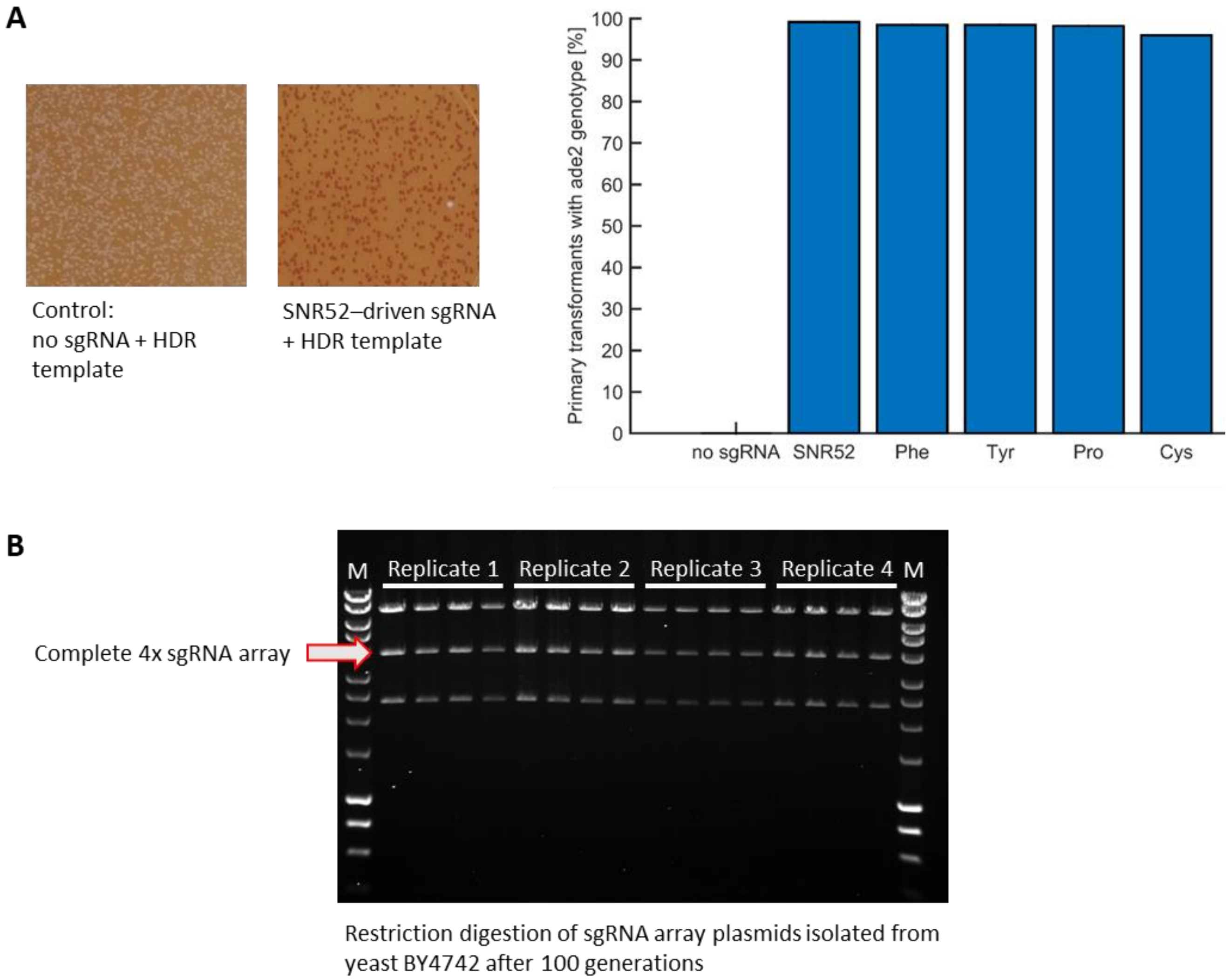

Supplementary Figure S1: Evaluation of the efficient expression and stable propagation of sgRNA cassettes. (**A**) Efficiency of individual sgRNA cassettes. The genome editing efficiency of individual sgRNA cassettes was tested by targeting the *ADE2* locus. Successful deletion results in red colonies with *ade2* genotype. The data shows editing efficiencies of *ADE2* targeting sgRNAs driven by the *SNR52* promoter compared to the indicated tRNA-driven expression cassettes. (**B**) Integrity of a 4x sgRNA array after plasmid propagation for >100 cell generations. A 4x sgRNA array was maintained in BY4742 cells for >100 generations. Plasmids rescued from the propagated cells were analysed by restriction digestion. The expected size of the complete 4x sgRNA array is indicated. M: 1 kb Hyper Ladder (Meridian Bioscience, Memphis, USA).

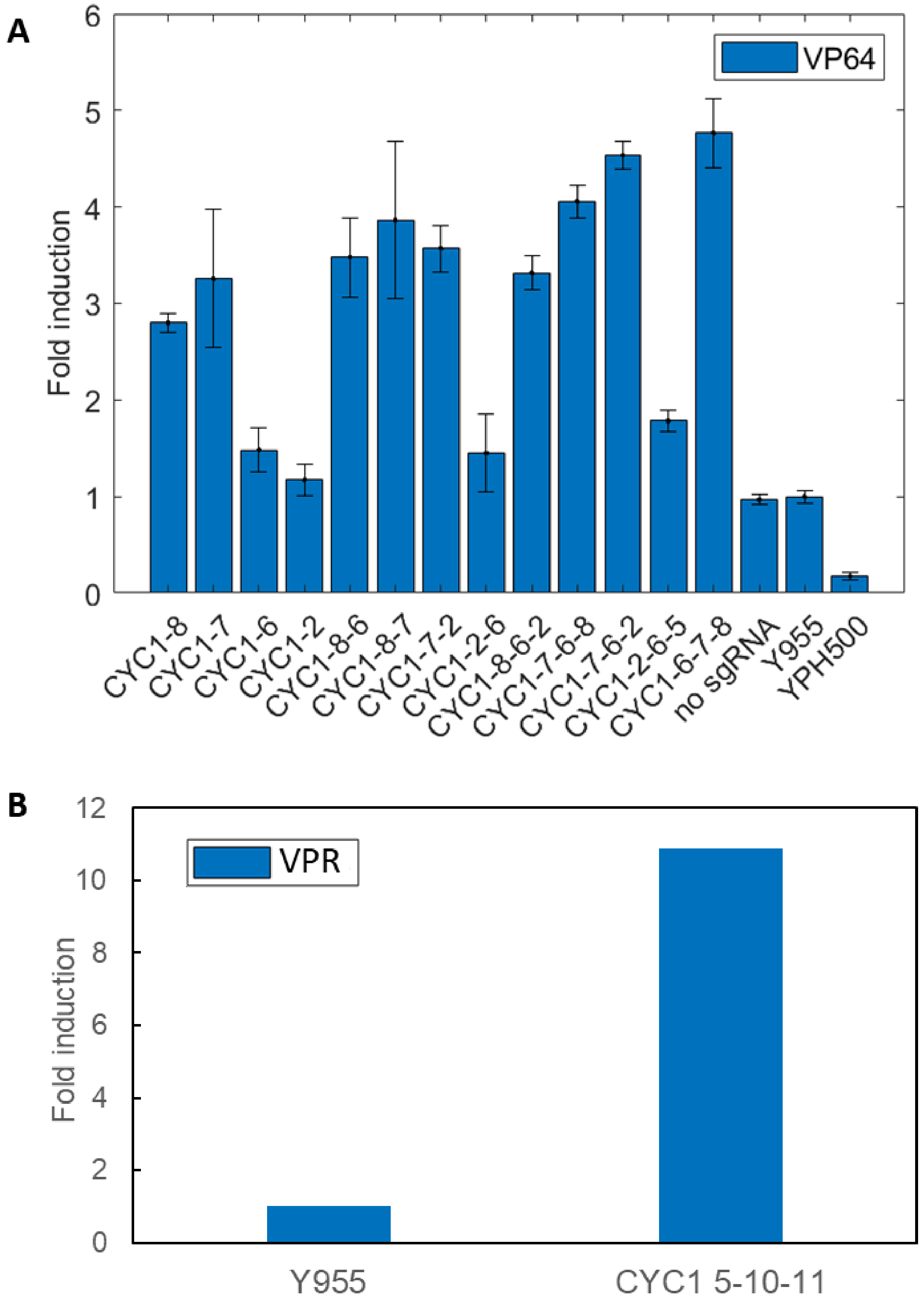

Supplementary Figure S2: Transactivation of *CYC1-yEGFP* expression mediated by rationally or randomly combined sgRNA arrays. (**A**) Rationally combined sgRNA arrays were tested for their ability to activate *CYC1-yEGFP* expression employing the VP64 AD. Error bars represent standard deviation calculated from three biologically independent experiments, each with three technical replicates. (**B**) The best performing randomly generated sgRNA array was selected via cell sorting for *CYC1-yEGFP* transactivation by the VPR AD. Results from a single experiment are shown.

A

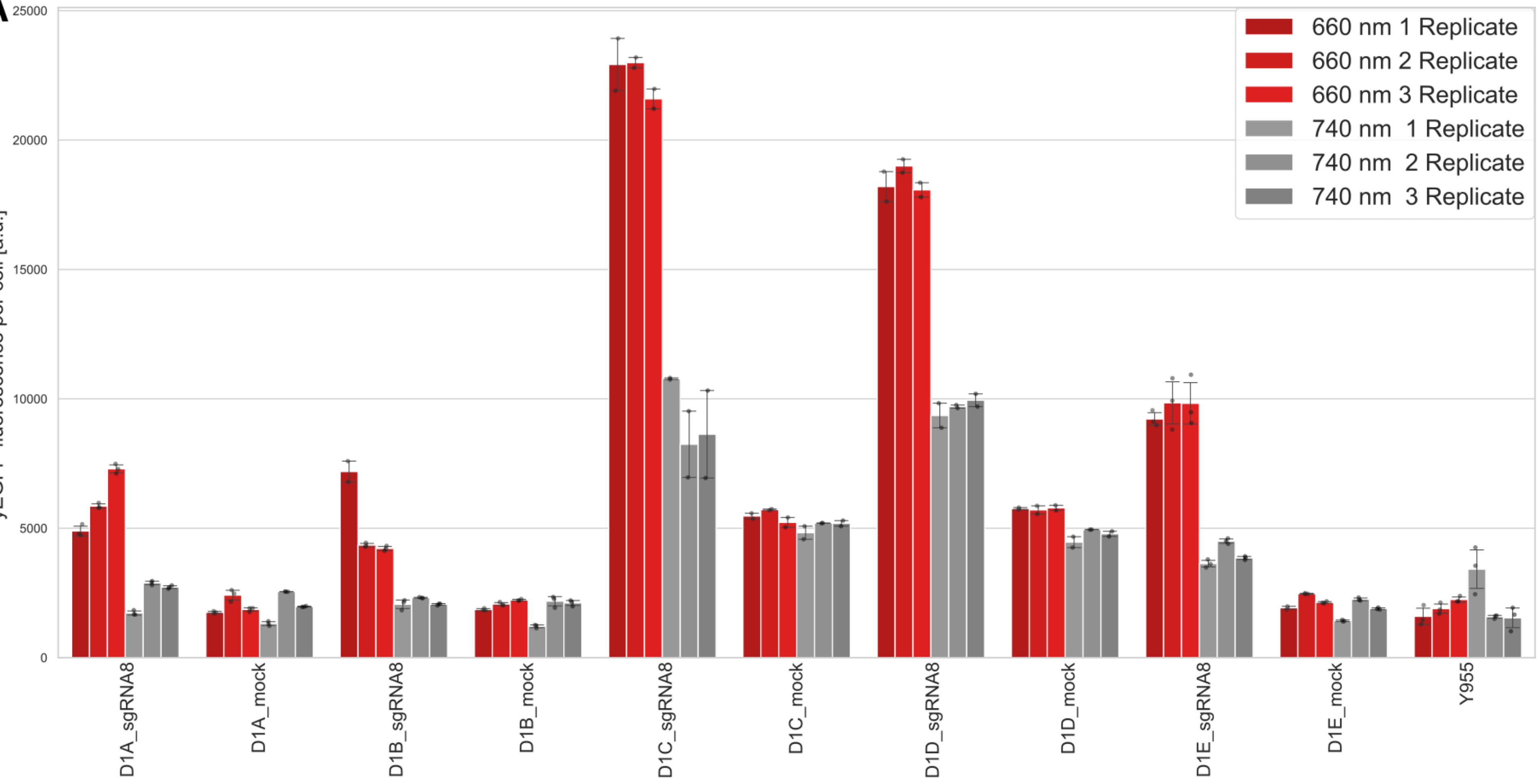
yEGFP-fluorescence per cell [a.u.]
0
5000
10000
15000
20000
25000
660 nm 1 Replicate
660 nm 2 Replicate
660 nm 3 Replicate
740 nm 1 Replicate
740 nm 2 Replicate
740 nm 3 Replicate
D1A_sgRNA8
D1A_mock
D1B_sgRNA8
D1B_mock
D1C_sgRNA8
D1C_mock
D1D_sgRNA8
D1D_mock
D1E_sgRNA8
D1E_mock
Y955

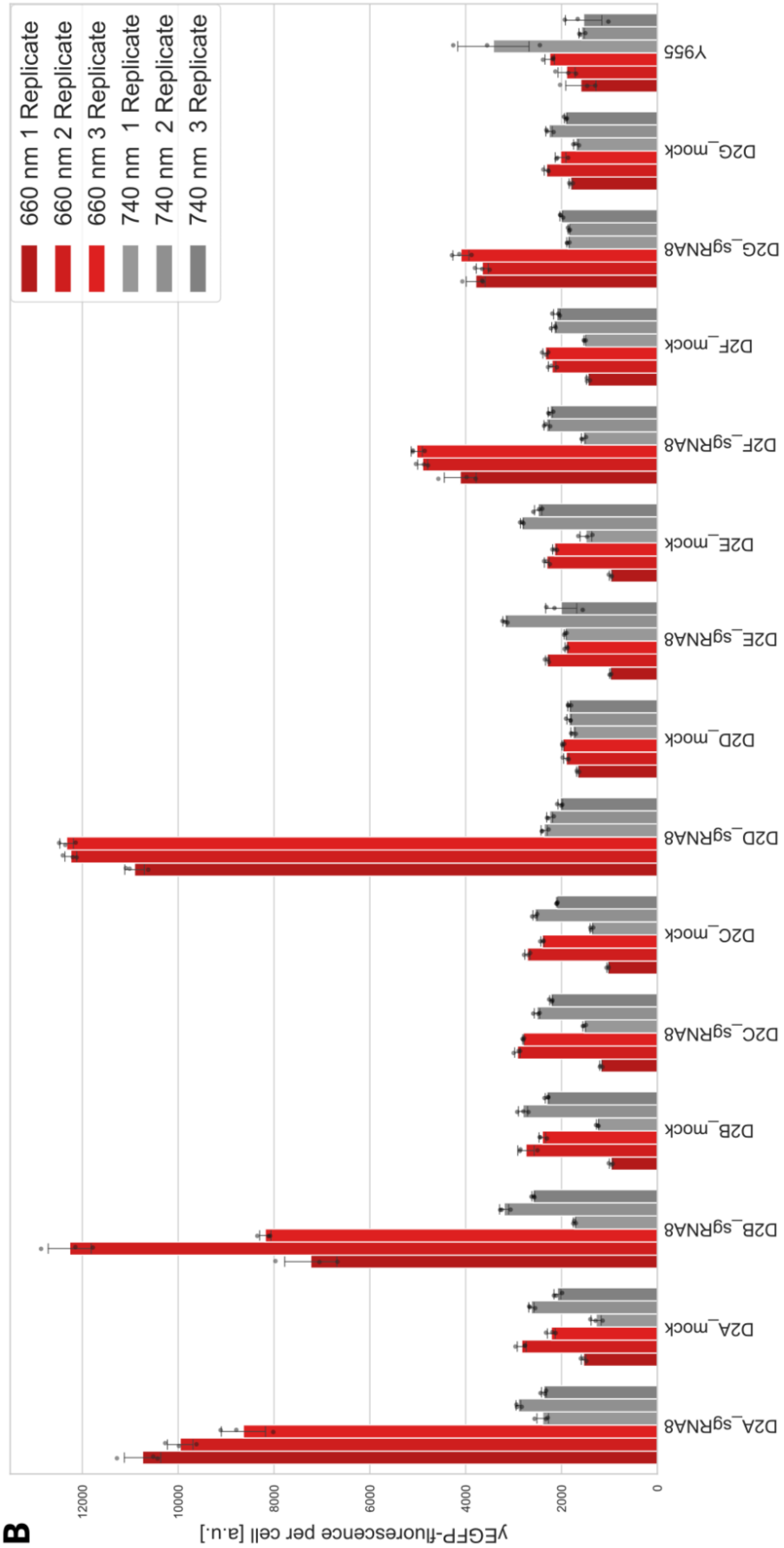

Supplementary Figure S3: Characterization of different designs of the light-inducible TF. Yeast strains holding a genome-integrated version of the indicated split TF and either sgRNA expression plasmid sgRNA8 (targeting the *CYC1* promoter) or a mock sgRNA were inoculated in 24-well plates in SD-Ura-Leu medium with 25 µM PCB to an OD_{600} of 0.1, treated with a single far-red light pulse and incubated in the dark, shaking at 30°C. After 6 h, induced samples were treated with a 30-sec red light pulse, followed by 10-sec red light pulses every 30 min for 16 h. Output of yEGFP-fluorescence was measured via flow cytometry and is given as geometrical mean of yEGFP fluorescence per cell, standard deviations are indicated as error bars. Each column represents a single biological replicate with three technical replicates each (indicated as grey dots). A.u.: arbitrary units. (**A**) Design 1, (**B**) Design 2.

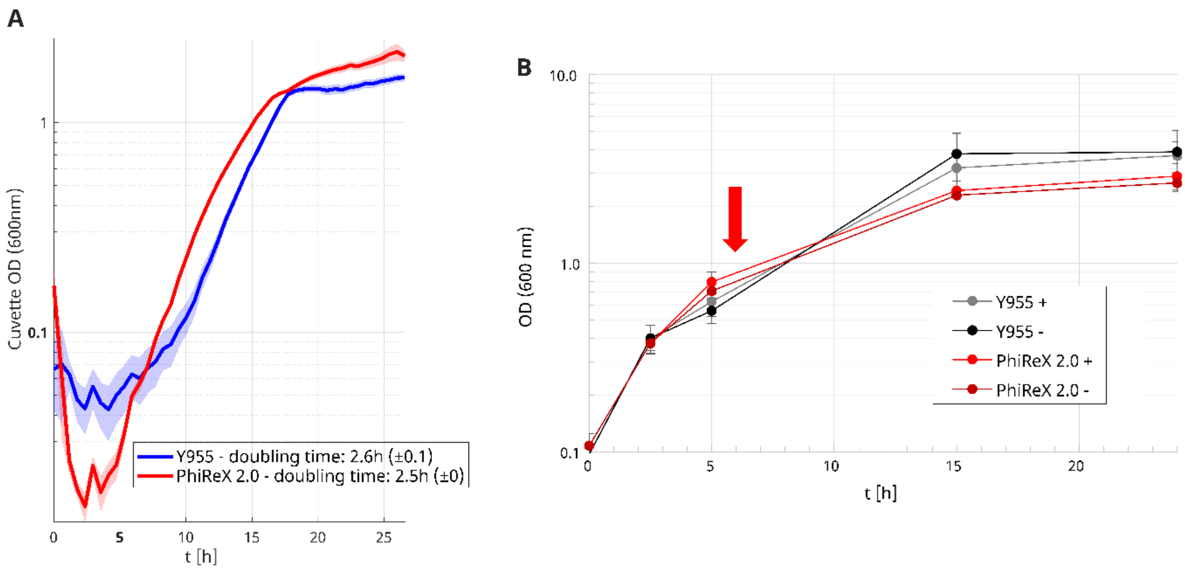

Supplementary Figure S4: Growth curve of PhiReX 2.0 compared to control strain Y955. (**A**) Strains Y955 and PhiReX 2.0 were pre-cultured in SD complete and SD-Ura, respectively, for 24 h. Cells were then washed and inoculated in fresh medium to OD 0.01 in 96-well plates and incubated shaking in a microplate reader. OD_{600} was recorded every 35 min. Shown is the mean from three biological replicates, each with three technical replicates. Standard deviation is indicated with shaded areas**.** (**B**) Strains Y955 (*CYC1-yEGFP* reporter) and PhiReX 2.0 were inoculated in 24-well plates in SD-Ura-Leu medium with 25 µM PCB to an OD_{600} of 0.1, treated with a single far-red light pulse and incubated shaking at 30°C. Samples marked with a plus were induced by a 30-sec red light pulse after 6 h, followed by 10-sec red light pulses every 30 min. Samples marked with a minus were kept in darkness for the whole incubation time. OD_{600} was measured 0 h, 2.5 h, 5 h, 15 h and 24 h after inoculation. Shown are the mean values and standard deviations calculated from three biological replicates, measured with two technical replicates each. The red arrow indicates the induction time point.

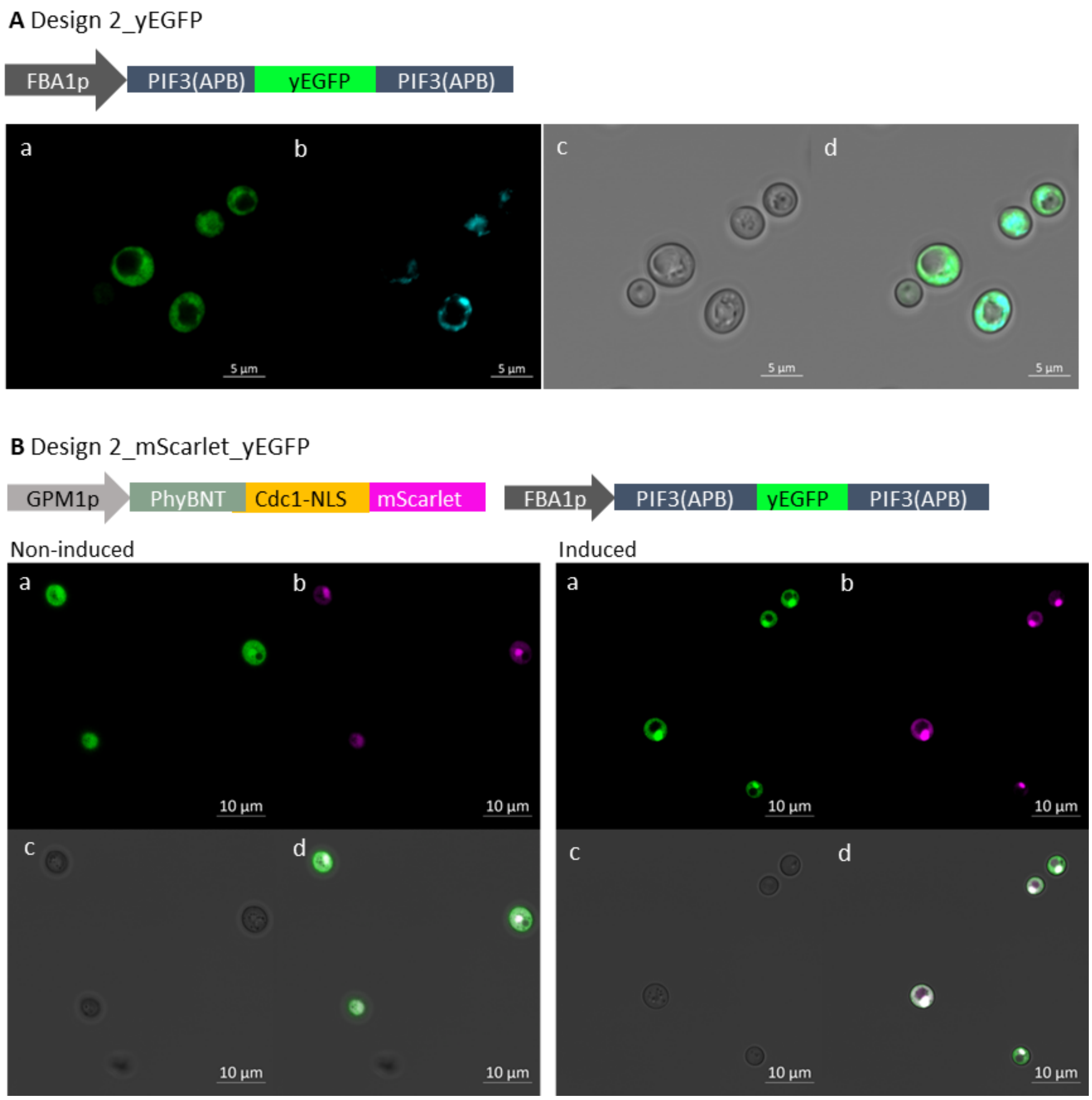

Supplementary Figure S5: Confocal microscopy analysis of the cellular localization of the optical dimer. (**A**) Yeast strain Design 2_yEGFP expresses the fluorescence reporter yEGFP with an N- and C-terminal fusion to PIF3(APB) under regulation of the *FBA1* promoter. The fusion protein is located in the cytoplasm. a: yEGFP fluorescence; b: DAPI fluorescence; c: bright field image; d: merge. (**B**) Yeast strain Design 2_mScarlet_yEGFP expresses two fusion proteins, the photoreceptor PhyBNT fused to the Cdc1-NLS, the fluorescent reporter mScarlet under the regulation of the *GPM1* promoter and yEGFP N- and C-terminally fused to PIF3(APB) under regulation of the *FBA1* promoter. In non-induced conditions, most of the yEGFP fusion protein is located in the cytoplasm with a small portion located in the nucleus. The mScarlet fusion protein carrying an NLS is clearly located in the nucleus. After light induction, both fluorescence reporters are mostly located in the nucleus with a small fraction available in the cytosol. a: yEGFP fluorescence; b: mScarlet fluorescence; c: bright field; d: merge.

Supplementary Table S1: Features of sgRNA multimerization vectors.

Plasmid	Cas9-variant	Auxotrophic selection marker	Dominant selection marker
pFM138	Cas9	*TRP*	None
pFM149	None	*URA*	None
pFM150	None	*URA*	None
pFM151	None	*URA*	None
pFM152	None	*URA*	None
pFM153	None	*URA*	None
pFM154	dCas9-VP64	*TRP*	$G418^R$
pFM155	dCas9-VP64	*TRP*	$Hygromycin^R$
pFM158	dCas9-VPR	*TRP*	$G418^R$
pFM159	dCas9-VPR	*TRP*	$Hygromycin^R$

Supplementary Table S2: Sequences of sgRNA target sites for *CYC1* with respective percentages of specificity and efficiency (on target) scores. Included is also the mock sgRNA, not binding within the yeast genome. n.a. = not applicable.

ID	Position relative to ATG	Strand	Sequence	PAM	Specificity score	Efficiency score	Primer for sgRNA cloning
Cyc1_1	-459	-1	TTCGACGATGTCGTCTCACA	CGG	99	60	FM1882, FM1883
Cyc1_2	-400	-1	CGGGAATTTTAGATTCAGGT	AGG	92	57	FM1884, FM1885
Cyc1_3	-354	-1	GCCAAAGAGACCGGAAAGAT	CGG	100	49	FM1886, FM1887
Cyc1_4	-332	-1	CTGCCATCGTCCGTAAACCC	CGG	100	62	FM1888, FM1889
Cyc1_5	-286	1	CCAGTTCATTTGGCGAGCGT	TGG	100	46	FM1890, FM1891
Cyc1_6	-261	1	GGTGGATCAAGCCCACGCGT	AGG	100	57	FM1892, FM1893
Cyc1_7	-219	1	CCAGGCGTGTATATATAGCG	TGG	78	71	FM1894, FM1895
Cyc1_8	-203	-1	GTGTCAGCACTAAAGTTGCC	TGG	100	56	FM1896, FM1897
Cyc1_9	-73	1	ATATTCTTTCCTTATACATT	AGG	89	34	FM1898, FM1899
Cyc1_10	-60	-1	TATAGTAATTTATGCTGCAA	AGG	83	56	FM1900, FM1901
Cyc1_11	-245	-1	ACGCCTGGCGGATCTGCTCG	AGG	100	59	FM1934, FM1935
Cyc1_12	-185	1	ACTTTAGTGCTGACACATAC	AGG	97	51	FM1936, FM1937
Cyc1_13	-148	1	TGCGACGACACATGATCATA	TGG	100	42	FM2141, FM2142
Mock	n.a.	n.a.	ATGTACAACAATATTTGTTA	n.a.	n.a.	n.a.	FM1745, FM1746

Supplementary Table S3: List of oligonucleotides used for cloning.

Name	Sequence
FM795	ATGTCTAAAGGTGAAGAATTATTCACTGGTGTTGTCCCAATTTTGGTTGAATTAGATGGTGATGTTAATGG
FM796	TTATTTGTACAATTCATCCATACCATGGGTAATACCAGCAGCAGTAACAAATTCTAACAAGACC
FM1745	gatcATGTACAACAATATTTGTTA
FM1746	aaacTAACAAATATTGTTGTACAT
FM1882	gatcTTCGACGATGTCGTCTCACA
FM1883	aaacTGTGAGACGACATCGTCGAA
FM1884	gatcCGGGAATTTTAGATTCAGGT
FM1885	aaacACCTGAATCTAAAATTCCCG
FM1886	gatcGCCAAAGAGACCGGAAAGAT
FM1887	aaacATCTTTCCGGTCTCTTTGGC
FM1888	gatcCTGCCATCGTCCGTAAACCC
FM1889	aaacGGGTTTACGGACGATGGCAG
FM1890	gatcCCAGTTCATTTGGCGAGCGT
FM1891	aaacACGCTCGCCAAATGAACTGG
FM1892	gatcGGTGGATCAAGCCCACGCGT
FM1893	aaacACGCGTGGGCTTGATCCACC
FM1894	gatcCCAGGCGTGTATATATAGCG
FM1895	aaacCGCTATATATACACGCCTGG
FM1896	gatcGTGTCAGCACTAAAGTTGCC
FM1897	aaacGGCAACTTTAGTGCTGACAC
FM1934	gatcACGCCTGGCGGATCTGCTCG
FM1935	aaacCGAGCAGATCCGCCAGGCGT
FM1936	gatcACTTTAGTGCTGACACATAC
FM1937	aaacGTATGTGTCAGCACTAAAGT
FM2141	gatcTGCGACGACACATGATCATA
FM2142	aaacTATGATCATGTGTCGTCGCA
GY036	CACTACGGATACTTTTACAACGGGAGCAGTTATTCAAGTTTATCATTATCAATACTGCCATTTCAAAG
GY037	CAGCGCGTCGGCGCGCCCTTTCTTACGCTTTAATGGCCTAGATTC
GY038	CCATTAAAGCGTAAGAAAGGGCGCGCCGACGCGCTGG
GY039	GATTTAAAGTAAATTCACTCATAACATATCGAGATCGAAATCGTCCAGAGC
GY040	GATCTCGATATGTTATGAGTGAATTTACTTTAAATCTTGCATT
GY041	TGTTTCACAACCAGAGATTCAGGATAGCACTTATTGTCATGTAGATGATACTGACTGCACGC
GY042	CACTACGGATACTTTTACAACGGGAGCAGTTATTCATAGTCGTGCAATGTATGACTTTAAGATTTG
GY043	CCCGACTCCGGAAACCATTATTGTAATATGTGTGTTTGTTTGGATTATTAAG
GY044	AACACACATATTACAATAATGGTTTCCGGAGTCGGG
GY045	ATCATTCATTCTTCAGACTCATAACATATCGAGATCGAAATCGTCCAGAGC
GY046	GATCTCGATATGTTATGAGTCTGAAGAATGAATGATTTGATGATTTCTTTTTCC
GY047	TGTTTCACAACCAGAGATTCAGGATAGCACTTATTGTATTCGAACTGCCCATTCAGC
GY048	CAATAAGTGCTATCCTGAATCTCTGGTTGTGAAACATCCAACTGGCACCGCTGGC
GY049	CTTATCCATTCCGCGGCCCACCTTACGTTTCTTTTTGGGGTGATGATGATGATTCAACCATGGAACA AAGTCATCG
GY050	AAAAAGAAACGTAAGGTGGGCCGCGGAATGGATAAGAAATACTCA
GY051	AAGCTCAAACAGAGGCATGGTGGCGCGCCCAACTTT
GY052	AAAGTTGGGCGCGCCACCATGCCTCTGTTTGAGCTTTTCAGGC

Name	Sequence
GY053	AATTAATTTGAATTAACTCTAATGATGATTCAACCATGGAACAAAGTCATCG
GY054	TGGTTGAATCATCATTAGAGTTAATTCAAATTAATTGA
GY055	TCTTGCATCTTACGATACCTGAGTATTCCCACAGTTAGTAAGCTACTATGAAAGA
GY056	CTTATCCATTCCGCGGCCCACCTTACGTTTCTTTTTGGGGTGATGCGACGATCCACAAAACTGATC AGAAGACCC
GY057	CAGCTAGGAGGTGACTAGAGTTAATTCAAATTAATTGA
GY059	GACCTTGGTGGTCGACATTTTGTTTGTTTATGTGTGTTTATTCGAAAC
GY060	CACACATAAACAAACAAAATGTCGACCACCAAGGTCACCT
GY061	CAGCGCGTCGGCGCGCCCTTTCTTACGCTTTAATGGCCTTTTCACACTGCCAGCATCATCTCTTTGAGC
GY062	GACCTTGGTGGTCGACATTATTGTAATATGTGTGTTTGTTTGGATTATTAAG
GY063	AACACACATATTACAATAATGTCGACCACCAAGGTCACCT
GY064	GGGAACGAGGTGACTCATTTTGAATATGTATTACTTGGTTATGGTTATATATGA
GY065	AAGTAATACATATTCAAAATGAGTCACCTCGTTCCCGACTGGG
GY066	CTTATCCATTCCGCGGCCCACCTTACGTTTCTTTTTGGGGTGATGTTGGGGGGCTCCACCGCC
GY067	AATTAATTTGAATTAACTCTAGTCACCTCCTAGCTGACTCAAATCAATGCG
GY117	CTTATCCATTCCGCGGCCGTGATGCGACGATCCACAAAACTG
GY118	TGTGGATCGTCGCATCACGGCCGCGGAATGGATAAGAAATACTCAATAGG
L1071	ATCCAAGGCTAGCGGGCGCGCCACCATGCCT
L1072	TCCGCGGCCGTGATGATGATGATTCAACCATGGAACAAAGTCA
L1073	GGTTGAATCATCATCATCACGGCCGCGGAATGGATAAG
L1074	GGCATGGTGGCGCGCCCGCTAGCCTTGGATCCGGC
L1075	CCATCTTCGACACATCTCTGTTTTGAGTCTGAAGAATGAATGATTTGATGATTTC
L1076	GGTCAGCCCTGCTGGCGCGCCCTTTCTTACG
L1077	GAAAGGGCGCGCCAGCAGGGCTGACCCCAAG
L1078	CATTCATTCTTCAGACTCAAAACAGAGATGTGTCGAAGATGGACAGT
L1100	GGATACTTTTACAACGGGAGCAGTTATTCACAACATAAATAATTTCTATTAACAATGTAATTTCCA
L1101	CTCCGGAAACCATTTCGAATTAGTTGTTTTGATGTGATAATAGAGGG
L1102	CAACTAATTCGAAATGGTTTCCGGAGTCGGGG
L1103	ATGCAAAATTTCTCATTTTCAATTCATAACATATCGAGATCGAAATCGTCCAGAGCA
L1104	TGAATTGAAAATGAGAAATTTTGCATAAAAAA
L1105	CCAGAGATTCAGGATAGCACTTATTGAATGATCGTGGAGTTTCAAACATCG
L1106	ATGCAAAATTTCTCATTTTCAATTCAAAACAGAGATGTGTCGAAGATGGACAGT
L1107	GCTATCCTGAATCTCTGGTTGTGAAACAAGCGTTGGTTGGTGGATCAAGCC
L1108	GCTCAAACAGAGGCATTATTAATTTAGTGTGTGTATTTGTGTTTGTGTGTC
L1109	ACACACACTAAATTAATAATGCCTCTGTTTGAGCTTTTCAGGC
L1110	AAAAGGGGCCTGTCTAATGATGATTCAACCATGGAACAAAGTCATCG
L1111	GAATCATCATTAGACAGGCCCCTTTTCCTTTGTCGA
L1112	CTTACGATACCTGAGTATTCCCACAGTTAGCTTGCAAATTAAAGCCTTCGAGCGT
L1235	GGTTGTGAAACAAACTGTGGGAATACTCAGGTATCG
L1237	ATGGTTTCTAAAGGTGAAGCAGTTATTAAGG
L1238	TCATTTGTATAATTCATCCATACCACCTG
L1239	GGTATGGATGAATTATACAAATGAGTCTGAAGAATGAATGATTTGATGATTTCTTTTTCC
L1240	CCTGAGTATTCCCACAGTTTGTTTCACAACCAGAGATTCAGG
L1241	GCTTCACCTTTAGAAACCATGGCGCGCCCTTTCTTACGCTTTAATGGCCTAGATTCT TTAAAAGAGTCTCTCAGAATAAGCTGG

Name	Sequence
L1243	TGGATGAATTGTACAAATAACCAATTGCCGGATCCAAGG
L1244	GCACTTATTGGCGGCCGCTGAATAACTGCTCCCGTTGTAAAAGTATCC
L1245	GCAGTTATTCAGCGGCCGCCAATAAGTGCTATCCTGAATCTCTGGTTG
L1247	AATTCTTCACCTTTAGACATTCCGCGGCCGTGATGATGATG
L1354	gatcACAACGCGTACAGTTTCACA
L1355	aaacTGTGAAACTGTACGCGTTGT
L1356	gatcCTACTTTTTACTATCTACGG
L1357	aaacCCGTAGATAGTAAAAAGTAG
L1358	gatcCAGTTCTGAATATCGCAGAA
L1359	aaacTTCTGCGATATTCAGAACTG
L1360	gatcAAGGGACAACTGGATATACA
L1361	aaacTGTATATCCAGTTGTCCCTT
L1362	gatcAGGGACAACTGGATATACAG
L1363	aaacCTGTATATCCAGTTGTCCCT
L1364	gatcCCTATGTATTACTATCACGT
L1365	aaacACGTGATAGTAATACATAGG
L1366	gatcTTTAGTTACATAAAATTCGG
L1367	aaacCCGAATTTTATGTAACTAAA
L1368	gatcCAGGGATAATAATAGCACAT
L1369	aaacATGTGCTATTATTATCCCTG
L1370	gatcACTATCGTATCTAATGACTG
L1371	aaacCAGTCATTAGATACGATAGT
L1380	gatcGATGATATGAGGAGGCATCG
L1381	aaacCGATGCCTCCTCATATCATC
L1382	gatcTGGTAGGAGGATAAATACAC
L1383	aaacGTGTATTTATCCTCCTACCA
L1384	gatcCTGTTCAATAGCAACAATGT
L1385	aaacACATTGTTGCTATTGAACAG
L1386	gatcAGTGGAATAGGATGGCGAGT
L1387	aaacACTCGCCATCCTATTCCACT
L1388	gatcATATCGACTCAAGCGACCAG
L1389	aaacCTGGTCGCTTGAGTCGATAT
L1390	gatcTGTAAAGTGCAATATTGCTG
L1391	aaacCAGCAATATTGCACTTTACA
CR_426	TTTGAATATGTATTACTTGGTTATGGTTATATATG
CR_427	GTTAATTCAAATTAATTGATATAGTTTTTTAATGAG
CR_428	ACCAAGTAATACATATTCAAAATGAGCAGGGCTGACCCC
CR_444	TTACAACGGGAGCAGTTATTCATCCAACTGGCACCGCTGG
CR_452	TGGGGTGATGAAACAGAGATGTGTCGAAGATGGAC
CR_453	CACATCTCTGTTTCATCACCCCAAAAAGAAACGTAAGG
CR_454	CCCTGCTCATGGTGGCGCGCCCAACTTTG
CR_455	GGCGCGCCACCATGAGCAGGGCTGACCCC
CR_456	ATCAATTAATTTGAATTAACTCTAAAACAGAGATGTGTCGAAGATGGAC
B01_r	TGAATAACTGCTCCCGTTGTAAAAG

Supplementary Table S4: Overview of Level 0 fragments and backbones used for the assembly of Level 1 plasmids.

Level 1 plasmid	Level 0 plasmids (HR)	Assembly components
pGY001	pGY005 (A0/A1)	TDH3p-PhyBNT-Cdc1NLS-VP64_AD-TDH3tt
	pGY007 (A1/A2)	pFBA1-PIF3(APB)-SV40NLS-dCas9-SV40NLS-PIF3(APB)-FBA1tt
	LEU marker (A2/C0)	
pGY002	pGY005 (A0/A1)	TDH3p-PhyBNT-Cdc1NLS-VP64_AD-TDH3tt
	pGY008 (A1/A2)	pFBA1-PIF3-SV40NLS-dCas9-FBA1tt
	LEU marker (A2/C0)	
pGY003	pGY006 (A0/A1)	GPM1p-PhyBNT-Cdc1NLS-VP64_AD-GPM1tt
	pGY007 (A1/A2)	FBA1p-PIF3(APB)-SV40NLS-dCas9-SV40NLS-PIF3(APB)-FBA1tt
	LEU marker (A2/C0)	
pGY004	pGY006 (A0/A1)	GPM1p-PhyBNT-Cdc1NLS-VP64_AD-GPM1tt
	pGY008 (A1/A2)	FBA1p-PIF3-SV40NLS-dCas9-FBA1tt
	LEU marker (A2/C0)	
pGY013	pGY009 (A0/A1)	TDH3p-MpPhy-Cdc1NLS-VP64_AD-TDH3tt
	pGY011 (A1/A2)	FBA1p-MpPIF-SV40NLS-dCas9-FBA1tt
	LEU marker (A2/C0)	
pGY014	pGY010 (A0/A1)	GPM1p-MpPhy-Cdc1NLS-VP64_AD-GPM1tt
	pGY011 (A1/A2)	FBA1p-MpPIF-SV40NLS-dCas9-FBA1tt
	LEU marker (A2/C0)	
pGY032	pGY005 (A0/A1)	TDH3p-PhyBNT-Cdc1NLS-VP64_AD-TDH3tt
	pGY028 (A1/A2)	FBA1p-PIF3-dCas9-FBA1tt
	LEU marker (A2/C0)	
pLH_114	pGY006 (A0/A1)	GPM1p-PhyBNT-Cdc1NLS-VP64_AD-GPM1tt
	pLH_113 (A1/A2)	FBA1p-PIF3(APB)-dCas9-PIF3(APB)-FBA1tt
	LEU marker (A2/C0)	
pLH_116	pLH_115 (A0/A1)	GPM1p-PhyBNT-Cdc1NLS-VPR_AD
	pGY007 (A1/A2)	FBA1p-PIF3(APB)-SV40NLS-dCas9-SV40NLS-PIF3(APB)-FBA1tt
	LEU marker (A2/C0)	
pLH_117	pLH_115 (A0/A1)	GPM1p-PhyBNT-Cdc1NLS-VPR_AD
	pLH_113 (A1/A2)	FBA1p-PIF3(APB)-dCas9-PIF3(APB)-FBA1tt
	LEU marker (A2/C0)	
pLH_134	pLH_129 (A0/A1)	RPL8ap-PhyBNT-Cdc1NLS-VP64_AD-RPL8att
	pGY007 (A1/A2)	pFBA1-PIF3(APB)-SV40NLS-dCas9-SV40NLS-PIF3(APB)-FBA1tt
	LEU marker (A2/C0)	
pLH_135	pLH_130 (A0/A1)	RPL8ap-PhyBNT-Cdc1NLS-VPR_AD-RPL8att
	pGY007 (A1/A2)	pFBA1-PIF3(APB)-SV40NLS-dCas9-SV40NLS-PIF3(APB)-FBA1tt
	LEU marker (A2/C0)	
pLH_204	pLH_201 (A0/A1)	GPM1p-PhyBNT-Cdc1NLS-mScarlet-GPM1tt
	pLH_202 (A1/A2)	FBA1p-PIF3(APB)-GFP-PIF3(APB)-FBA1tt
	LEU marker (A2/C0)	

Level 1 plasmid	Level 0 plasmids (HR)	Assembly components
pCR_110	pLH_116	B01_r/CR_427
	pLH_116	CR_444/CR_426
	pLH_116	CR_428/CR_452
	pLH_116	CR_453/CR_454
	pLH_116	CR_455/CR_456

Supplementary Table S5: Overview of the PCR templates and PCR primers used for the assembly of Level 0 plasmids.

Level 0 plasmid	PCR template	PCR primer
pGY005	pRL_CT	GY036/GY037
	pcDNA3.1-Cry2FL-VP64	GY038/GY039
	pRL_CT	GY040/GY041
	pL0B_0-1 (*Hin*dIII)	
pGY006	pPC_012	GY042/GY043
	pRL_CT	GY044/GY037
	pcDNA3.1-Cry2FL-VP64	GY038/GY045
	pPC_012	GY046/GY047
	pL0B_0-1 (*Hin*dIII)	
pGY007	pRL_CT	GY048/GY049
	pcDNA3.1-CibN-dCas9-CibN	GY050/GY051
	pRL_CT	GY052/GY053
	pRL_CT	GY054/GY055
	pL0B_1-R (*Hin*dIII)	
pGY008	pRL_CT	GY048/GY056
	pcDNA3.1-CibN-dCas9-CibN	GY050/GY067
	pRL_CT	GY057/GY055
	pRL_CT	GY036/GY059
	pL0B_1-R (*Hin*dIII)	
pGY009	*Marchantia polymorpha* gDNA	GY060/GY061
	pcDNA3.1-Cry2FL-VP64	GY038/GY039
	pRL_CT	GY040/GY041
	pL0B_0-1 (*Hin*dIII)	
pGY010	pPC_012	GY042/GY062
	Marchantia polymorpha gDNA	GY063/GY061
	pcDNA3.1-Cry2FL-VP64	GY038/GY045
	pPC_012	GY046/GY047
	pL0B_0-1 (*Hin*dIII)	
pGY011	pRL_CT	GY048/GY064
	Marchantia polymorpha gDNA	GY065/GY066
	pcDNA3.1-CibN-dCas9-CibN	GY050/GY067
	pRL_CT	GY057/GY055
	pL0B_1-R (*Hin*dIII)	
pGY028	pGY008	GY0048/GY0117
	pGY008	GY0118/GY0055
pLH_113	pGY007	L1071/L1072
	pGY007	L1073/L1074
pLH_115	pGY006	L1075/L1076
	pFM159	L1077/L1078
pLH_129	pPC_032	L1100/L1101
	pGY006	L1102/L1103
	pPC_032	L1104/L1105
	pL0B_0-1 (*Hin*dIII)	
pLH_130	pPC_032	L1100/L1101
	pLH_115	L1102/L1106
	pPC_032	L1104/L1105
	pL0B_0-1 (*Hin*dIII)	

Level 0 plasmid	PCR template	PCR primer
pLH_201	pLH_114	L1235/L1241
	mScarlet	L1237/L1238
	pLH_114	L1239/L1240
pLH_202	DNA898	FM795/FM796
	pLH_114	L1245/L1247
	pGY003	L1243/L1244

Supplementary Table S6: List of yeast strains generated in this study.

**Name**	**Precursor strain**	**Light system (*ura3-52*)**	**sgRNA (episomal)**
Y955	YPH500		
Y1094	YPH500	pLH_116	
Design 1_A_sgRNA8	Y955	pGY002	sgRNA8
Design 1_A_mock	Y955	pGY002	mock
Design 1_B_ sgRNA8	Y955	pGY004	sgRNA8
Design 1_B_mock	Y955	pGY004	mock
Design 1_C_ sgRNA8	Y955	pGY013	sgRNA8
Design 1_C_mock	Y955	pGY013	mock
Design 1_D_ sgRNA8	Y955	pGY014	sgRNA8
Design 1_D_mock	Y955	pGY014	mock
Design 1_E_ sgRNA8	Y955	pGY032	sgRNA8
Design 1_E_mock	Y955	pGY032	mock
Design 2_A_ sgRNA8	Y955	pGY001	sgRNA8
Design 2_A_mock	Y955	pGY001	mock
Design 2_B_ sgRNA8	Y955	pGY003	sgRNA8
Design 2_B_mock	Y955	pGY003	mock
Design 2_C_ sgRNA8	Y955	pLH_134	sgRNA8
Design 2_C_mock	Y955	pLH_134	mock
**Design 2_D_sgRNA8 (PhiReX 2.0)**	Y955	pLH_116	sgRNA8
Design 2_D_mock	Y955	pLH_116	mock
Design 2_E_ sgRNA8	Y955	pLH_135	sgRNA8
Design 2_E_mock	Y955	pLH_135	mock
Design 2_F_ sgRNA8	Y955	pLH_114	sgRNA8
Design 2_F_mock	Y955	pLH_114	mock
Design 2_G_ sgRNA8	Y955	pLH_117	sgRNA8
Design 2G_mock	Y955	pLH_117	mock
Design 2_ yEGFP	YPH500	pLH_202	
Design 2_mScarlet_yEGFP	YPH500	pLH_204	
VPR-SV40-dCAS9-SV40 VPR	YPH500	pCR_110	sgRNA8
pADH1	YPH500	pCR_094	
pCYC1	YPH500	pAT09	
